# The molecular basis for recognition of bacterial ligands at equine TLR2, TLR1 and TLR6

**DOI:** 10.1186/1297-9716-44-50

**Published:** 2013-07-04

**Authors:** Katherine Lucy Irvine, Lee Jason Hopkins, Monique Gangloff, Clare Elizabeth Bryant

**Affiliations:** 1 Department of Veterinary Medicine, University of Cambridge, Madingley Road, Cambridge CB30ES, UK; 2 Department of Biochemistry, University of Cambridge, Tennis Court Road, Cambridge CB21QW, UK

## Abstract

TLR2 recognises bacterial lipopeptides and lipoteichoic acid, and forms heterodimers with TLR1 or TLR6. TLR2 is relatively well characterised in mice and humans, with published crystal structures of human TLR2/1/Pam3CSK4 and murine TLR2/6/Pam2CSK4. Equine TLR4 is activated by a different panel of ligands to human and murine TLR4, but less is known about species differences at TLR2. We therefore cloned equine TLR2, TLR1 and TLR6, which showed over 80% sequence identity with these receptors from other mammals, and performed a structure-function analysis. TLR2/1 and TLR2/6 from both horses and humans dose-dependently responded to lipoteichoic acid from *Staphylococcus aureus*, with no significant species difference in EC50 at either receptor pair. The EC50 of Pam2CSK4 was the same for equine and human TLR2/6, indicating amino acid differences between the two species’ TLRs do not significantly affect ligand recognition. Species differences were seen between the responses to Pam2CSK4 and Pam3CSK4 at TLR2/1. Human TLR2/1, as expected, responded to Pam3CSK4 with greater potency and efficacy than Pam2CSK4. At equine TLR2/1, however, Pam3CSK4 was less potent than Pam2CSK4, with both ligands having similar efficacies. Molecular modelling indicates that the majority of non-conserved ligand-interacting residues are at the periphery of the TLR2 binding pocket and in the ligand peptide-interacting regions, which may cause subtle effects on ligand positioning. These results suggest that there are potentially important species differences in recognition of lipopeptides by TLR2/1, which may affect how the horse deals with bacterial infections.

## Introduction

Pathogen-associated molecular pattern recognition receptors (PRRs) on host cells detect pathogens and their associated ligands to initiate innate immune responses and drive inflammation. Toll-like receptors (TLRs) are the best characterised of the PRRs, and recognise predominantly bacterial and/or viral ligands. Bacterial lipoproteins, lipoteichoic acid (LTA) and peptidoglycan (PepG), are recognised by TLR2, which forms heterodimers with either TLR1 or TLR6 [1-3]. In mice and humans, the TLR2/1 heterodimer recognises triacylated lipopeptides, such as N-palmitoyl-S-[2,3-bis(palmitoyloxy)-propyl]-(R)-cysteinyl-(lysyl)3-lysine (Pam3CSK4), whereas the TLR2/6 complex recognises diacylated lipopeptides, for example S-[2,3-bis(palmitoyloxy)-propyl]-(R)-cysteinyl-(lysyl)3-lysine (Pam2CSK4) [1,2,4]. The TLR2/1 heterodimer also recognises diacylated ligands such as macrophage-activating lipopeptide 2 (MALP-2) and Pam2CSK4 [4]. Crystal structures for human TLR2/1 bound to Pam3CSK4 and murine TLR2/6 bound to Pam2CSK4 have been solved, and demonstrate the same heterodimeric structure with 1:1:1 TLR2:TLR1/6:ligand stoichiometry [1,2].

TLR2 knockout mice are hyper-susceptible to Gram positive bacterial infections, but resistant to Gram positive bacterial ligands, and humans with mutations in the TLR2 gene have altered Gram positive infection susceptibility [5-7]. TLR2 recognition of Gram positive bacteria is, therefore, fundamental to successfully controlling infections with these organisms. Gram positive bacterial infections in young horses are common, and involved in a range of syndromes from reduced performance in young racing thoroughbreds to life-threatening sepsis in neonates [8,9]. Equine peripheral blood mononuclear cells (PBMC) stimulated with the TLR2/1 ligand Pam3CSK4 produce tumour necrosis factor-α (TNF-α), and LTA and PepG also stimulate release of TNF-α in equine whole blood assays [10,11]. These findings suggest TLR2 recognition of bacterial ligands is important in the horse, but do not provide clues regarding how ligands interact with the binding pockets of equine TLR2/1 and TLR2/6 heterodimers, which is important for targeting future therapeutics. TLR2 antagonists could be useful for the treatment of Gram positive bacterial disease, and agonists useful as potential vaccine adjuvants.

Species differences in ligand recognition at TLRs are common, and, at TLR4/MD-2 for example, the underlying molecular mechanisms are becoming clearer [12]. Equine, human and murine TLR4/MD-2 show distinct profiles of ligand recognition, for example signalling to lipid IVa, and comparative molecular studies have been important in understanding how different ligands generate active signalling complexes [13,14]. Much less is known about ligand specificity at TLR2 in complex with co-receptors TLR1 or TLR6 in species other than humans and mice, however.

In this study, we cloned equine TLR2, TLR1 and TLR6. We performed a structure-function analysis to determine whether the equine receptors respond to classical human/murine TLR2 ligands, and with the same pharmacological phenotype as the human receptors. Our results demonstrate that subtle structural and functional species differences exist in recognition of lipopeptides by the TLR2/1 heterodimer. Pam3CSK4 responses were diminished at equine TLR2/1, which suggests the horse could have a dampened response to triacylated lipopeptides in general. This may have implications for therapeutic manipulation of equine TLR2, and could potentially indicate altered susceptibility to specific bacterial infections.

## Materials and methods

### Cells

Total RNA was isolated from either an equine macrophage line (EML) [15] or primary equine vascular smooth muscle (VSM). VSM cells were cultured from aorta removed *post mortem* from a horse euthanased at the Queen’s Veterinary School Hospital, University of Cambridge on welfare grounds, and for reasons unrelated to this study. SW620 cells were a gift from Richard Tapping and maintained in RPMI (Sigma-Aldrich, Poole, UK) supplemented with 10% foetal calf serum (FCS; Hyclone, Thermo Scientific, Cramlington, UK), 2 mM glutamine, 100 IU/mL penicillin and 100 μg/mL streptomycin (referred to as “complete SW620 medium”) [4]. HEK293 cells were purchased from ATCC and maintained in Dulbecco’s Modified Eagle medium (DMEM; Sigma-Aldrich) supplemented with 10% FCS, 2 mM l-glutamine, 100 IU/mL penicillin and 100 μg/mL streptomycin (referred to as “complete HEK medium”). Cells were grown at 37°C/5% CO_2_ and subcultured every 2-3 days.

### RNA isolation

Total RNA was isolated from cells using the RNeasy plus mini-kit (Qiagen, Manchester, UK). RNA yield and integrity were assessed by spectrophotometry and agarose gel electrophoresis (AGE) respectively.

### RT-PCR and cloning of full length equine TLR1, -2 and -6

Primers for amplification of equine TLR2, -1 and -6 by reverse transcriptase PCR (RT-PCR) were based on the published full-length cloned or predicted equine sequences. Primer sequences included restriction sites at the 5′ and 3′ termini to facilitate ligation into pcDNA3. First-strand cDNA was synthesised using the High Fidelity Transcriptor cDNA Synthesis Kit (Roche, Welwyn Garden City, UK) as follows: 1 μg of total RNA was incubated with 10 μM gene-specific reverse primer at 65°C for 5 min; reverse transcription master-mix comprising 5× Reaction Buffer, 10 mM dNTP, RNase inhibitor and High Fidelity Reverse Transcriptase (RT) was added to the denatured RNA, and mixtures incubated at 42°C for 1 h; RT enzyme was then inactivated by heating at 70°C for 10 min. First-strand cDNA was stored at -80°C or amplified by PCR using Phusion High-Fidelity (HF) DNA Polymerase (Thermo Scientific), according to the following recipe: 18.25 μL nuclease-free water, 2.5 μL 5× Phusion HF buffer, 0.5 μL 10 mM dNTP, 1.25 μL 10 μM forward primer, 1.25 μL 10 μM reverse primer, 2 μL first-strand cDNA template and 0.25 μL Phusion HF DNA Polymerase. The PCR protocol involved an initial denaturation step at 98°C for 3 min, followed by 34 cycles of denaturation at 98°C for 30 s, annealing at 55°C for 30 s, extension at 72°C for 1 min 30 s, and a final elongation step at 72°C for 10 min. PCR products of 2.5 kb, 2.6 kb and 2.4 kb, corresponding to full length equine TLR2, TLR1 and TLR6 respectively, were obtained by AGE and purified by gel extraction using the GeneJET gel extraction kit (Fermentas, Thermo Scientific). A-tailed PCR products were ligated into pGEM-T Easy Vector (Promega, Southampton, UK) and sequenced by conventional Sanger sequencing. Cloned sequences were compared with published sequences for equine TLR2 [GenBank:AY429602], TLR1 [GenBank:NM_001256899] and TLR6 [Genbank:NM_001257142], as appropriate, to verify the correct product was obtained. Sequenced products were then subcloned into pcDNA3 expression vector for functional studies.

### RT-PCR for detection of human TLRs

Internal primers for detection of human TLRs were designed using PrimerBLAST. Total RNA isolated from SW620 and HEK293 cells was subjected to DNase (Fermentas, Thermo Scientific) treatment to remove contaminating genomic DNA (gDNA) as follows: 8 μL (800 ng) total RNA was incubated with 1 μL DNaseI buffer and 1 μL DNaseI for 30 min at 37°C; 1 μL RNase-free EDTA was added and mixtures heated for 10 min at 65°C to inactivate the DNase. First-strand cDNA was then synthesised in a two-step process, using the SuperScript III First-Strand Synthesis System SuperMix (Invitrogen, Life Technologies, Paisley, UK): 6 μL DNase-treated total RNA was incubated with 1 μL 50 mM oligo dT and 1 μL 8× Annealing Buffer for 5 min at 65°C, then placed on ice; 10 μL 2× First-Strand Reaction Mix and 2 μL Superscript III/RNaseOUT Enzyme Mix were added and mixtures incubated at 50°C for 50 min. RNase was then inactivated by heating to 85°C for 5 min. cDNA was amplified by PCR using DreamTaq polymerase (Fermentas, Thermo Scientific) and gene-specific primers according to the following recipe: 12.88 μL nuclease-free water, 2.5 μL 10× DreamTaq buffer, 0.5 μL 10 mM dNTP, 0.5 μL 10 μM forward primer, 0.5 μL 10 μM reverse primer, 8 μL DNase-treated first-strand cDNA template and 0.125 μL DreamTaq DNA Polymerase. The PCR protocol involved an initial denaturation step at 95°C for 2 min, followed by 25-35 cycles of denaturation at 95°C for 30 s, annealing for 30 s, extension at 72°C for 50 s, and a final elongation step at 72°C for 15 min. Primer annealing temperatures were defined in preliminary optimisation experiments as follows: TLR4: 62.1°C; TLR2 and TLR1: 63.9°C; TLR6: 58.5°C; glyceraldehyde 3-phosphate dehydrogenase (GAPDH): 70.1°C. Negative control PCR reactions used DNase-treated total RNA instead of DNase-treated first-strand cDNA template, using an equal quantity of total RNA for fair comparison. Expression of TLRs was determined by AGE.

### Structural modelling

Three-dimensional structure images were generated using PyMol. The human TLR2/1 heterodimer crystal structure was downloaded from the Protein Data Bank [PDB:2Z7X]. A structural model of human TLR6 was generated using SWISS-MODEL, with the murine TLR6 crystal structure as a template [PDB:3A79] [16-19]. Structural models of the equine receptors were generated in the same way, using crystal structures of human TLR2, human TLR1 and murine TLR6. The human TLR1 TIR image was generated from the crystal structure [PDB:1FYV]. Pairwise protein sequence alignments of human and equine TLR1, -2 and -6 were generated with ClustalW Local Alignment tool. Variant residues within our cloned equine receptors were identified following alignment with published predicted and cloned equine sequences in ClustalW Pairwise Nucleotide alignment. Sequence alignments between human, equine and murine receptors were generated using ClustalW Multiple Sequence Alignment tool, and using standard settings.

### Bacterial ligands

Racemic mixtures of Pam2CSK4 and Pam3CSK4, and purified LTA from *Staphylococcus aureus*, were purchased from Invivogen, Source BioScience, Cambridge, UK.

### Transient transfection and stimulation of SW620 cells

SW620 cells (a gift from Richard Tapping, USA) were utilised for TLR2/1 and TLR2/6 transient transfection experiments, as, unlike HEK293 cells, they are 1) deficient in significant expression of all three receptors, and 2) poorly responsive to Pam2CSK4 and Pam3CSK4 without exogenously added TLRs (Additional file 1) [4]. This cell line also does not express CD14, which is required for efficient recognition of lipopeptides and LTA by TLR2 but does not alter the pattern of agonism displayed by synthetic ligands [4,20,21]. SW620 cells were seeded at 3.75 × 10^4^ cells per well of a 48 well plate and transiently transfected 24 hours later using Fugene 6 (Promega) at a ratio of 1 μg DNA: 4 μL Fugene 6. For transfection, expression vectors containing cDNA encoding human (in pFLAG-CMV) or equine (in pcDNA3) TLR2, -1 and -6, and CD14 (in pcDNA3), were mixed as appropriate. TLR cDNA-containing vectors were used at 2.5 ng/well; CD14 cDNA-containing vectors were used at 1.25 ng/well. TLR constructs were replaced with empty vector (pcDNA3), as appropriate, to balance total added DNA amounts. Reporter constructs pNF-κB-luc (Clontech; 25 ng/well) and ph-RG-TK (Promega; 12.5 ng/well), encoding firefly luciferase under an NF-κB promoter and constitutively expressed *Renilla* luciferase respectively, were then added to all receptor mixtures, together with 10× Tris-EDTA (TE). Following mixing of DNA, and per 4 wells, 0.7 μL Fugene 6 was diluted to 10 μL in serum- and antibiotic-free RPMI and incubated for 5 min. DNA mixtures were then added to the Fugene 6/RPMI mixtures and incubated for 30 min at room temperature. Following incubation, transfection mixtures were diluted to 1 mL per 4 wells in complete SW620 medium. Old medium was removed from plated cells and replaced with the transfection/complete medium mixtures. Twenty four hours post-transfection, cells were stimulated with either bacterial ligands diluted in RPMI containing 0.1% FCS (to provide a source of lipopolysaccharide binding protein (LBP)), or this medium alone as a control. Old media were removed before the addition of stimulation media. Cells were stimulated for six hours, lysed using diluted passive lysis buffer (PLB; Promega), and NF-κB activation measured by the dual luciferase reporter assay (Promega).

### Transient transfection and stimulation of HEK293 cells

HEK293 cells were seeded at a density of 3 × 10^4^ cells per well of a 96 well plate and transiently transfected 48 h later using JetPEI (Polyplus). For transfection, expression vectors containing cDNA encoding human TLR2, -1 and -6, and CD14, were mixed as appropriate. TLR cDNA-containing and CD14 cDNA-containing vectors were used at 1 ng/well. TLR constructs were replaced as appropriate with empty vector (pcDNA3) to balance the total added DNA amounts. pNF-κB-luc (10 ng/well) and ph-RG-TK (5 ng/well) were added to receptor mixtures, together with 10× TE and 150 mM NaCl to give a total volume of 50 μL per 10 wells. Following mixing of DNA, and per 10 wells, 2 μL JetPEI was added to 48 μL 150 mM NaCl. JetPEI/NaCl was added to the DNA mixtures at a 1:1 ratio and incubated for 30 min at room temperature. Following incubation, transfection mixtures were diluted to 1 mL per 10 wells in complete HEK medium. Old medium was removed from plated cells and replaced with the transfection/complete medium mixtures. Forty eight hours post-transfection, cells were stimulated with either bacterial ligands diluted in DMEM containing 0.1% FCS (to provide LBP), or this medium alone as a control. Old media were removed before the addition of stimulation media. Cells were stimulated for six hours, lysed using diluted PLB, and NF-κB activation measured by the dual luciferase reporter assay (Promega).

### Statistical analysis

Experiments were undertaken three times (*n* = 3) to ensure qualitative repeatability, and results shown from a representative experiment [4,13,22]. Multiple comparisons were performed using unpaired two-tailed t-tests with Bonferroni correction, and with Welch’s correction for unequal variances as appropriate. Dose response curves were fitted by non-linear regression to a sigmoidal dose-response (variable slope) model, using GraphPad Prism software, to allow determination of EC50 and the associated 95% confidence intervals (CI). For comparison of curves, best fit values for maximum stimulation and logEC50 were compared using F tests.

## Results

### Equine TLR2, -1 and -6 share high sequence identities with the human and murine receptors

Total RNA was harvested from equine cells and used to generate cDNA encoding equine TLR2, -1 and -6 by RT-PCR. Comparison of our constructs with the human receptor sequences revealed 81%, 81% and 80% whole receptor sequence identities respectively (Additional file 2). The ectodomains of TLR2, -1 and -6 were the least conserved regions (78%, 79% and 77% respectively), whereas the TIR domains were the most conserved (93%, 89% and 91% respectively). Full-length sequence alignments are shown in Additional file 3.

Within the GenBank database, equine TLR2 and TLR1 sequences are available from cloned receptors, whereas the sequence for equine TLR6 is predicted. On comparison of our cloned receptors with the published sequences, several variations were present. All changes were found to be non-synonymous. The ultra-high fidelity enzyme Phusion HF was used for amplification of single-strand cDNA sequences, and so cloning errors due to low enzyme specificity are unlikely. The sequence differences therefore probably reflect true population variability. To determine the potential functional significance of the variant residues, models of the equine receptors were generated based on crystal structures for the human and murine receptors, and variant residues mapped to show their location relative to ligand-binding and dimerisation sites [1,2,16-19].

One variant residue, L773W, was found in equine TLR1 (residues are labelled as changes from published equine → our cloned equine receptors). This residue is located in the TIR domain, well away from the BB loop, and represents a change from a small to bulkier non-polar residue (Additional file 4A). The equivalent W769 is present within human TLR1. A variant (V118L) that is present in a published partial equine TLR1 cDNA was not found in our construct [GenBank: EF581164.1]. The variations within equine TLR2 (R357Q, K509P and H579R) are all equivalent to the human or murine residues. None of these residues are located immediately adjacent to the binding pocket or the main dimerisation interface, and are therefore unlikely to interact with the ligand or interfere with dimerisation (Additional file 4B). Three variations are present within equine TLR6 (V452I, V486A and L580S), and located well away from the binding pockets (Additional file 4C). Only variant residues close to the dimerisation interfaces are shown for TLR2 and TLR6.

### *Staphylococcus aureus* LTA activates equine and human TLR2 heterodimers with similar potency

LTA is a major component of the Gram positive cell wall, and its recognition by TLR2 is probably a key initiator of Gram positive sepsis in humans [23,24]. Crystallography so far has shown binding of an inactive form of LTA from *Streptococcus pneumoniae* (pnLTA) to murine TLR2, but the dimeric structure of TLR2 bound to biologically active LTA has not been solved [1]. It is also unclear whether LTA signals through TLR2/1, TLR2/6 or both. We therefore constructed dose response curves for equine and human TLR2/1 and TLR2/6 to purified LTA from *Staphylococcus aureus*, using the human SW620 cell line. SW620 cells do not signal to TLR2 ligands, and respond very poorly when transfected with human TLR2 alone (Additional file 1C). Reconstitution of the TLR2/1 and TLR2/6 dimers is therefore required for robust signalling by this cell line to TLR2 ligands, which allows species-specific TLR2 dimer responses to be studied more reliably than in HEK293 cells.

**Figure 1:**
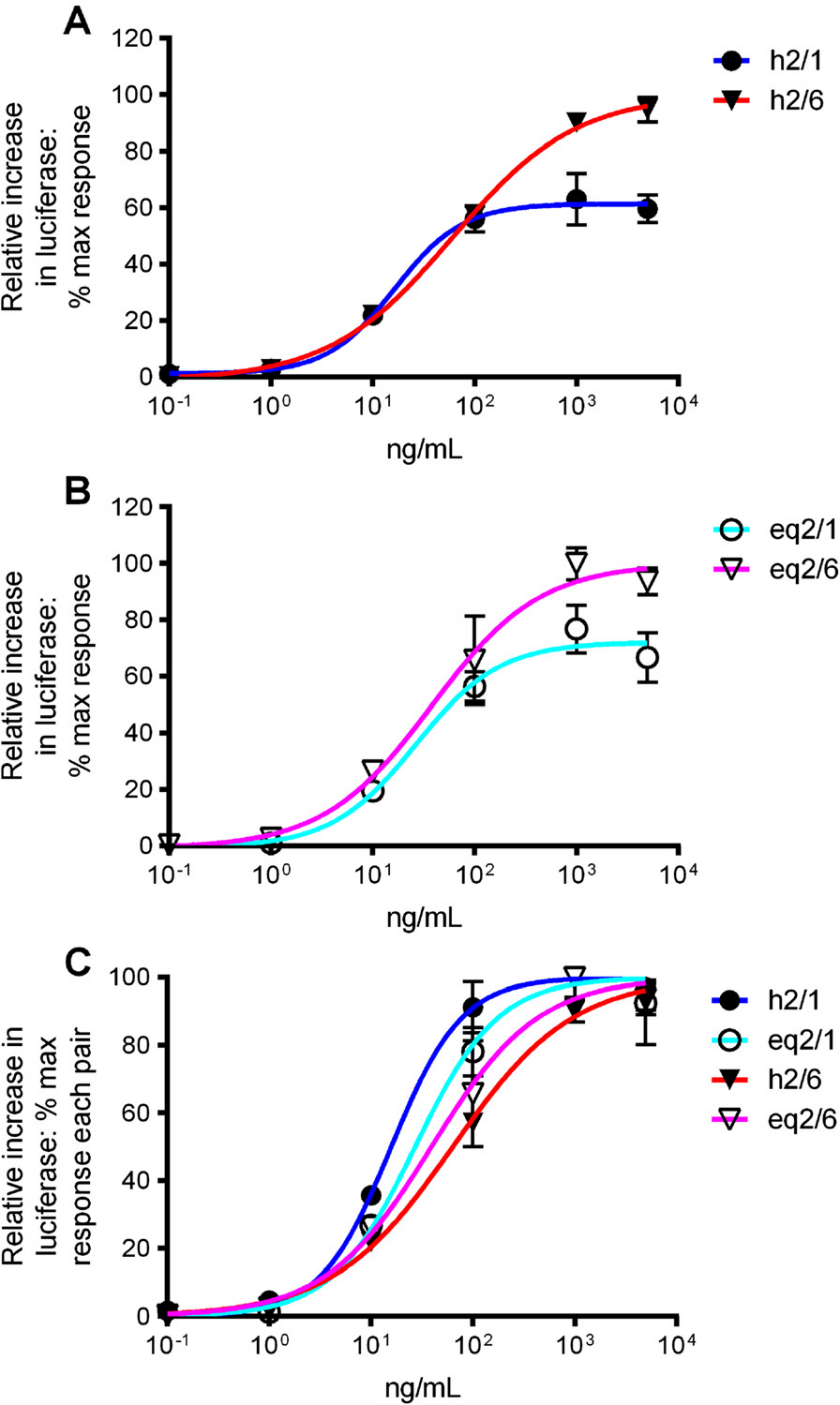
**Responses of human and equine TLR2/1 and TLR2/6 to LTA.** SW620 cells were transiently transfected with human TLR2 (hTLR2) and human TLR1 (hTLR1), hTLR2 and human TLR6 (hTLR6), equine TLR2 (eqTLR2) and equine TLR1 (eqTLR1), or eqTLR2 and equine TLR6 (eqTLR6), together with the cognate species’ CD14 and reporter constructs NF-κB-luc and *Renilla* luciferase. Cells were stimulated 24 hours later with increasing doses of LTA from *Staphylococcus aureus.* Cells were stimulated for six hours, lysed, and analysed for luciferase activity. Data are from a representative experiment and expressed as triplicate mean ± standard error of the mean (SEM) for that experiment. **(A)** LTA dose-dependently stimulated human TLR2/1 and TLR2/6. Maximum stimulation of human TLR2/6 was higher than human TLR2/1. **(B)** LTA dose-dependently stimulated equine TLR2/1 and equine TLR2/6. Maximum stimulation of equine TLR2/6 was higher than TLR2/1. **(C)** Data for each curve were normalised for calculation of EC50, using medium alone as 0% and maxima for each curve (raw data) from **(A)** and **(B)** as 100%. EC50 values were not significantly different for human and equine receptor pairs, nor between equine receptor pairs. Human TLR2/1 and TLR2/6 had significantly different EC50 values.

SW620 cells were transiently transfected with reporter constructs and one of four receptor combinations: human TLR2+TLR1+CD14, human TLR2+TLR6+CD14, equine TLR2+TLR1+CD14 or equine TLR2+TLR6+CD14, and stimulated with increasing doses of ligand. EC50 and maximum stimulation values were determined at each heterodimer as measures of ligand potency and efficacy respectively. LTA efficacy was significantly higher at TLR2/6 than TLR2/1 for both species’ heterodimers (Figure 1A and 1B). The EC50 for LTA at human TLR2/1 (16.82 ng/mL (95% C.I. 9.82-26.49)) was significantly lower than that at human TLR2/6 (63.59 ng/mL (95% C.I. 51.47-78.56)), whereas potency at the equine TLR2 heterodimers was not significantly different (equine TLR2/1 EC50 = 27.11 ng/mL (95% C.I. 16.22-48.29); equine TLR2/6 EC50 = 39.32 ng/mL (95% C.I. 22.31-69.29); Figure 1C). The calculated EC50 values were also not significantly different between species for LTA at TLR2/1 or at TLR2/6. These findings show that *S. aureus* LTA activates both TLR2/1 and TLR2/6 analogously in the two species, with no clear species-specific behaviour.

### Species differences are present between equine and human TLR2/1 responses to Pam2CSK4 and Pam3CSK4

**Figure 2:**
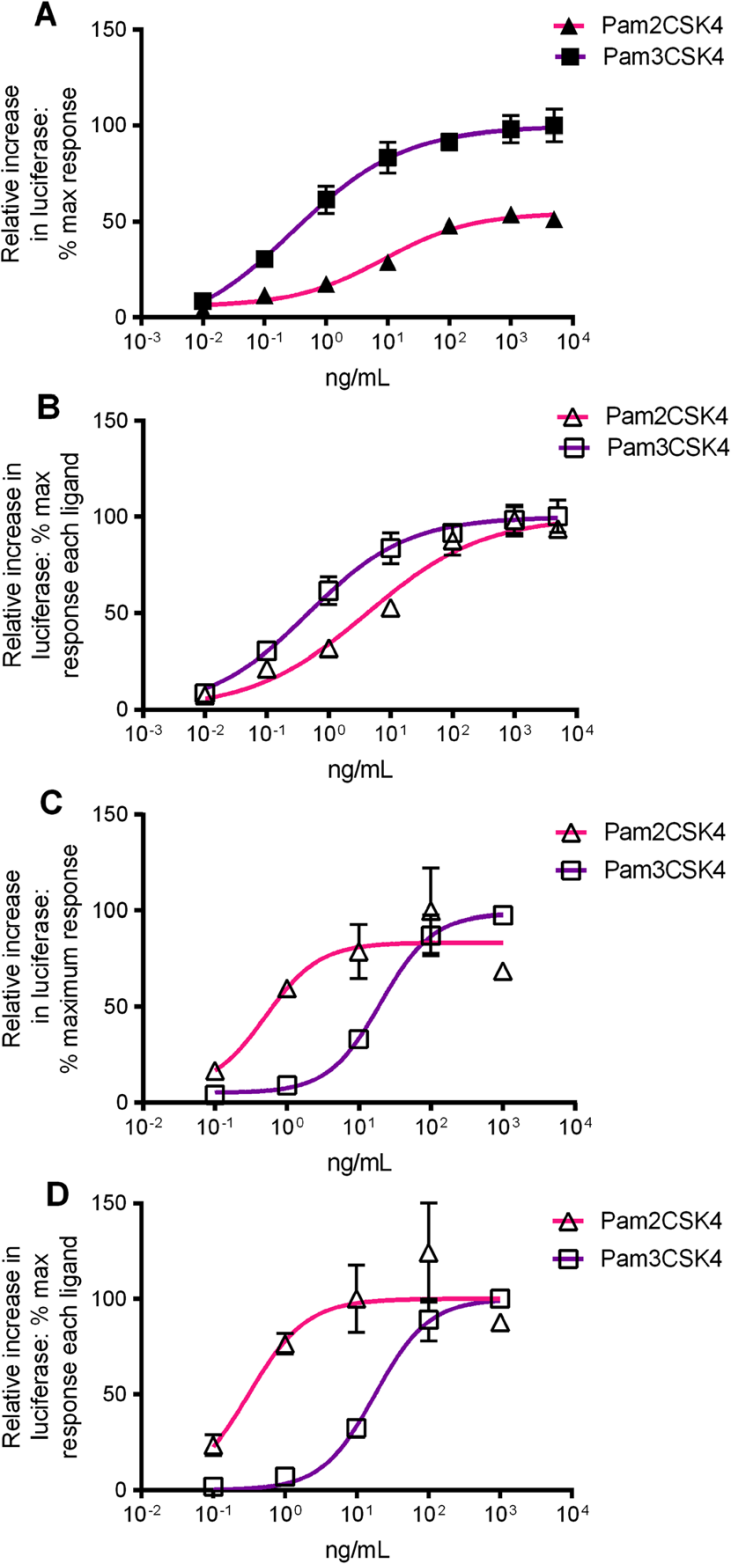
**Responses of human and equine TLR2/1 to synthetic ligands.** SW620 cells were transiently transfected with hTLR2 and hTLR1 or eqTLR2 and eqTLR1, together with the cognate species’ CD14 and reporter constructs NF-κB-luc and *Renilla* luciferase. Cells were stimulated 24 hours later with increasing doses of synthetic ligands Pam2CSK4 and Pam3CSK4. Cells were stimulated for six hours, lysed, and analysed for luciferase activity. Data are from a representative experiment and expressed as triplicate mean ± SEM for that experiment. **(A)** Pam3CSK4 and Pam2CSK4 stimulated human TLR2/1 dose-dependently, and maximum stimulation by Pam3CSK4 was higher than for Pam2CSK4. **(B)** Data from **(A)** were normalised for calculation of EC50, using medium alone as 0% and maxima for each curve (raw data) from **(A)** as 100%. EC50 values were significantly different for the two ligands, with the Pam2CSK4 curve shifted to the right relative to Pam3CSK4. **(C)** Pam2CSK4 and Pam3CSK4 stimulated equine TLR2/1 dose-dependently, and maximum stimulation by both ligands was the same. **(D)** Data from **(C)** were normalised for calculation of EC50, using medium alone as 0% and maxima for each curve (raw data) from **(C)** as 100%. The curve for Pam3CSK4 was shifted to the right, and EC50 values were significantly different.

Pam2CSK4 and Pam3CSK4 are synthetic lipopeptides that are diacylated and triacylated respectively [3]. The availability of crystal structures for murine TLR2/6/Pam2CSK4 and human TLR2/1/Pam3CSK4 makes these ligands excellent candidates for structure-function analysis. We constructed dose responses curves to Pam2CSK4 or Pam3CSK4 in transfected SW620 cells. Pam2CSK4 and Pam3CSK4, as expected, stimulated human TLR2/1 dose-dependently, with higher efficacy observed for Pam3CSK4 at this receptor heterodimer (Figure 2A). The EC50 values for Pam2CSK4 and Pam3CSK4 at human TLR2/1 were significantly different, with Pam2CSK4 having an EC50 of 4.21 ng/mL (95% confidence interval (C.I.) 2.34-7.50) and Pam3CSK4 an EC50 of 0.47 ng/mL (95% C.I. 0.28-0.79) (Figure 2B). Pam3CSK4 is therefore stronger than Pam2CSK4 at human TLR2/1. Dose response curves for Pam2CSK4 and Pam3CSK4 at equine TLR2/1 demonstrated two key differences to the curves obtained for human TLR2/1. Firstly, Pam2CSK4 and Pam3CSK4 efficacies at equine TLR2/1 were the same (Figure 2C). Secondly, the EC50 for Pam3CSK4 (18.73 ng/mL (95% C.I. 12.95-27.08)) was higher than for Pam2CSK4 (0.43 ng/mL (95% C.I. 0.13-1.48)) (Figure 2D) which is in contrast to the results for human TLR2/1. Pam3CSK4 is therefore less potent than Pam2CSK4 at equine TLR2/1. Pam2CSK4 was also 10-fold more potent at equine TLR2/1 than at human TLR2/1. These findings demonstrate TLR2/1 recognition of these synthetic lipopeptides is somewhat different between the horse and human.

**Figure 3:**
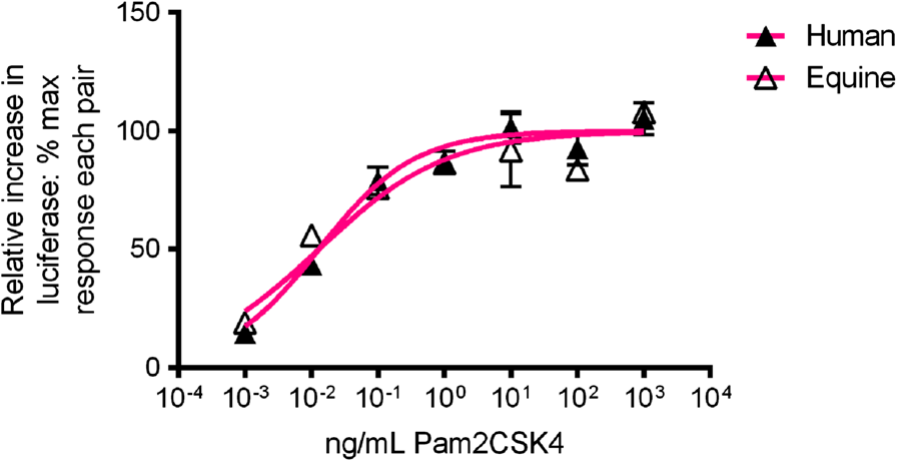
**Responses of human and equine TLR2/6 to Pam2CSK4.** SW620 cells were transiently transfected with hTLR2 and hTLR6 or eqTLR2 and eqTLR6, together with the cognate species’ CD14 and reporter constructs NF-κB-luc and *Renilla* luciferase. Cells were stimulated 24 hours later with increasing doses of Pam2CSK4. Cells were stimulated for six hours, lysed, and analysed for luciferase activity. Data are from a representative experiment and expressed as triplicate mean ± SEM for that experiment. Pam2CSK4 stimulated human and equine TLR2/6 dose-dependently. Data were normalised for calculation of EC50, using medium alone as 0% and maxima for each curve (raw data) as 100%. EC50 values were not significantly different for human and equine receptors.

The EC50 values for Pam2CSK4 at human TLR2/6 (0.015 ng/mL (95% C.I. 0.009-0.027)) and equine TLR2/6 (0.010 ng/mL (95% C.I. 0.004-0.024)) were not significantly different (Figure 3). Pam3CSK4 failed to stimulate TLR2/6 from either species (data not shown). These findings show Pam2CSK4 recognition by equine TLR2/6 is analogous to human TLR2/6 recognition.

### Comparison between equine and human TLR2/1 structures reveals potential differences at the ligand interaction interfaces

**Figure 4:**
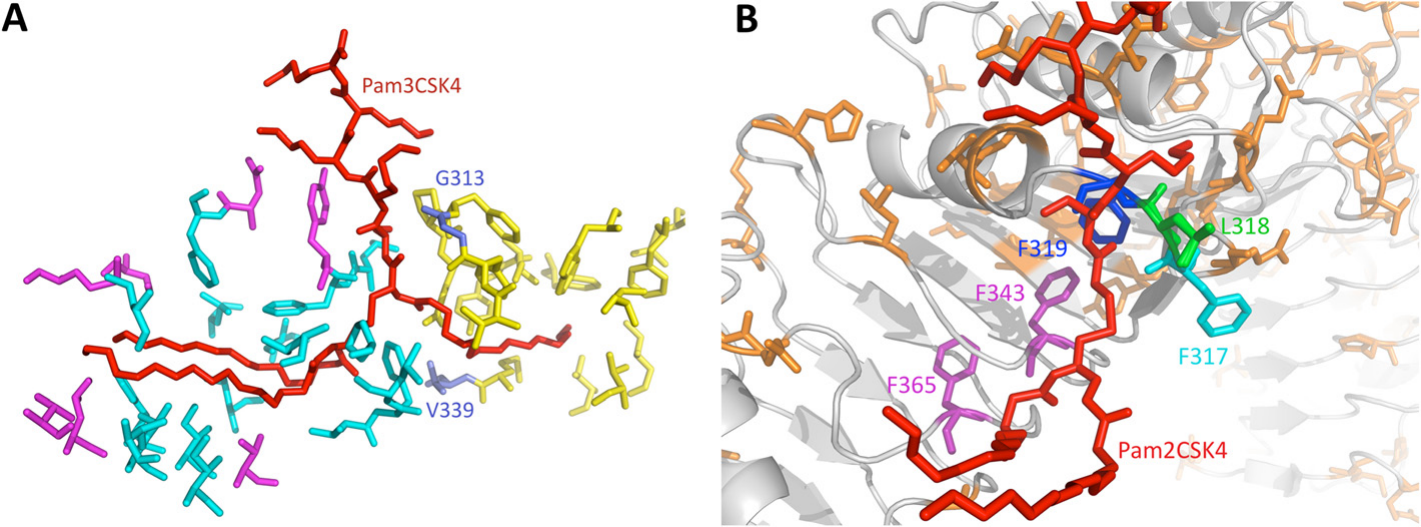
**Species-differences of the ligand binding pockets of TLR2/1.** Crystal structures of human TLR2 and TLR1 [PDB code 2Z7X], and a model of human TLR6, were viewed in PyMol and non-conserved residues between the human and horse highlighted. **(A)** Conserved TLR2 ligand-binding residues (cyan) form the majority of contacts, whereas non-conserved (magenta) are at the periphery of the pocket. The TLR1 pocket is lined with conserved residues (yellow); only G313 and V339 (lilac; S317 and I343 in the horse) are non-conserved. **(B)** L318 (green) and F319 (blue), which interact with Pam2CSK4 (red), are conserved in the horse (I318 and F319). The region surrounding L318 and F319 is conserved apart from F317 (Y317 in the horse; cyan), which lies immediately lateral to L318. F343 and F365 (magenta), which block the TLR6 pocket, are also shown.

Residues within human TLR2/1 responsible for ligand binding and dimerisation have been identified [2]. We compared the equine and human receptors to determine whether non-conservative changes between the two species’ sequences are located in areas of the proteins that could affect ligand association, and explain species specificity at TLR2/1. Human-equine non-conserved residues are spread diffusely across both receptor ectodomains (data not shown). The binding pockets of TLR2 and TLR1 are lined predominantly with human-equine conserved residues, whereas the regions of the two receptors flanking the ligand peptide group are virtually all non-conserved (Additional file 5). Seven of the twenty four residues of human TLR2 that interact with Pam3CSK4 in the crystal structure are non-conserved between human and equine, but these lie mainly on the periphery of the ligand binding pocket (Figure 4A). All but three of the TLR dimerisation residues of TLR2 are conserved between the two species, and all of the residues that interact with both the ligand and form the dimerisation interface are also conserved (not shown). Two of the eighteen residues in human TLR1 that interact with Pam3CSK4 are non-conserved between the two species (Figure 4A), and all but four of the main dimerisation interface residues are conserved (not shown). Of the two non-conserved residues, only V339 (I343 in the equine) contacts the amide acyl chain of Pam3CSK4. In summary, nine ligand-binding and seven dimerisation residues differ between the equine and human TLR2/1 dimers, which could potentially explain the small species differences in ligand recognition at this receptor pair.

In the mouse TLR2/6/Pam2CSK4 crystal structure, two residues only (L318 and F319) within TLR6 are shown to interact directly with Pam2CSK4 [1]. The shallow binding pocket of human TLR6 is flanked and lined by human-equine conserved residues, except for F317 (Y317 in the horse), which lies immediately lateral to L318 (I318 in the horse) and forms part of the main dimerisation interface (Figure 4B). Given the equipotency of Pam2CSK4 and LTA at horse and human TLR2/6, the effect of this residue change appears negligible. The two phenylalanines, F343 and F365, that block the entrance to the TLR6 pocket and prevent entry of an acyl chain are conserved between human, horse and mouse (Additional file 6).

### Species differences in TLR2/1 signalling are dependent on both TLR2 and TLR1

**Figure 5:**
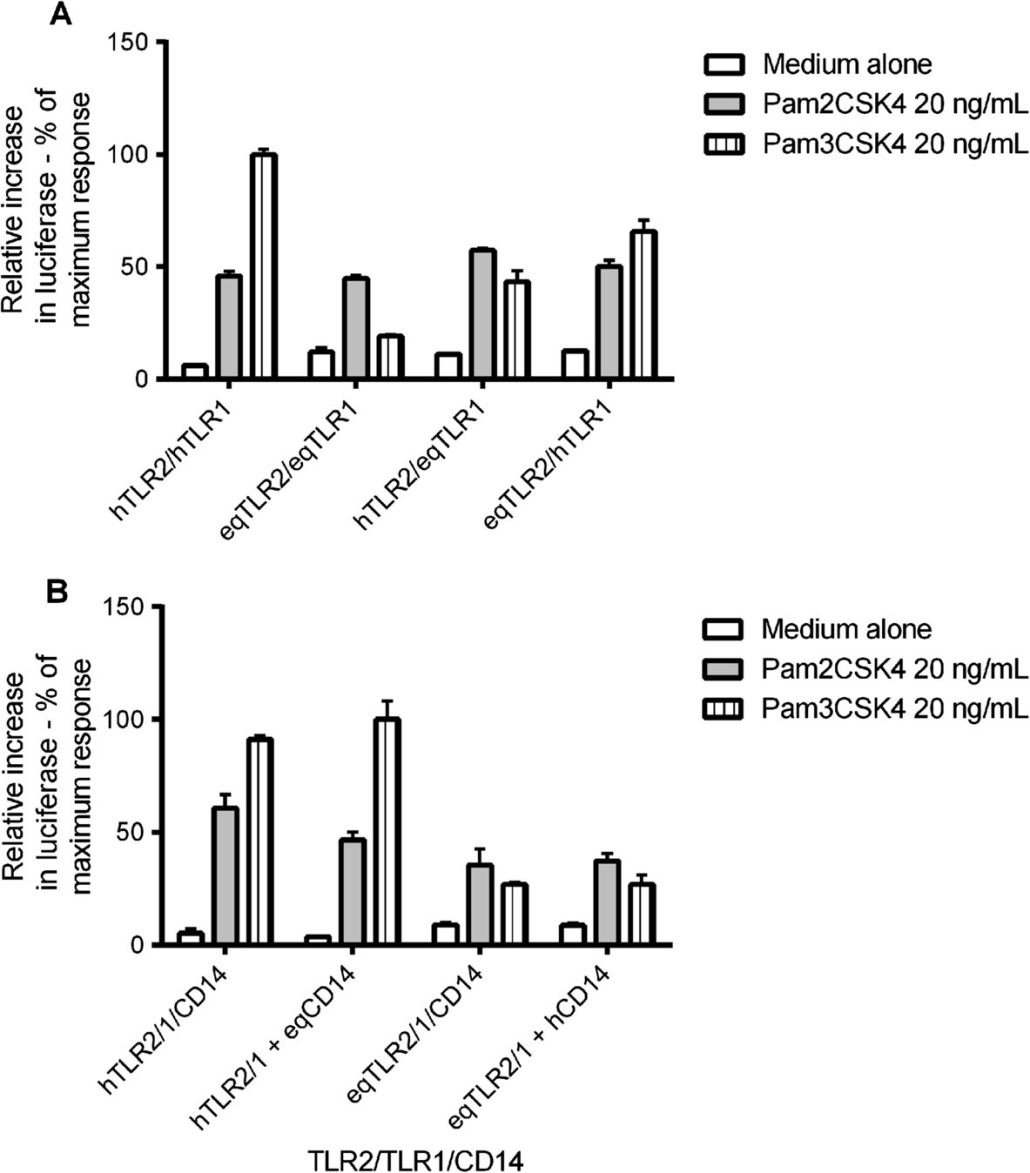
**Effect of swapping species of TLR1 and CD14 on Pam2CSK4/Pam3CSK4 stimulation.** SW620 cells were transiently transfected with combinations of TLR2, TLR1 and CD14 (as indicated in the graphs) and reporter constructs NF-κB-luc and *Renilla* luciferase. Cells were stimulated 24 hours later with Pam2CSK4 at 20 ng/mL, Pam3CSK4 at 20 ng/mL, or medium alone. Cells were stimulated for six hours, lysed, and analysed for luciferase activity. Data are from a representative experiment and expressed as triplicate mean ± SEM for that experiment. **(A)** Swapping the species of TLR1 does not restore the equine response to Pam2CSK4/Pam3CSK4 to that of human. **(B)** Swapping the species of CD14 did not affect responses to Pam2CSK4 or Pam3CSK4.

The amino acid residues identified by structural modelling suggested both TLR2 and TLR1 could be required for the species differences seen in the lipopeptide responses. To test this hypothesis, we transfected SW620 cells with human TLR2+TLR1+CD14, equine TLR2+TLR1+CD14, human TLR2+CD14+equine TLR1, or equine TLR2+CD14+human TLR1. Cells were stimulated with Pam2CSK4 at 20 ng/mL, Pam3CSK4 at 20 ng/mL, or medium alone. A ligand concentration of 20 ng/mL was selected from our preliminary dose response curve analyses and comparison with published data, as this dose induces submaximal responses for both ligands [4,25]. The Pam3CSK4 response was higher than Pam2CSK4 at human TLR2/1, yet lower than Pam2CSK4 at equine TLR2/1, consistent with the dose response curves (Figure 5A). Human TLR2/equine TLR1 and equine TLR2/human TLR1 each signalled similarly to both ligands, however, with no statistically significant difference in signal between ligands detected at either receptor combination. Replacing equine TLR2 or TLR1 with the equivalent human receptor therefore did not replicate the human TLR2/1 response pattern to these ligands, which indicates residues in both equine TLR2 and equine TLR1 contribute to the observed equine TLR2/1 responses to Pam2CSK4 and Pam3CSK4.

To ensure that the observed species differences to Pam2CSK4 or Pam3CSK4 were not caused by species specificity at CD14, SW620 cells were transiently transfected with one of the following four receptor combinations: human TLR2+TLR1+CD14, human TLR2+TLR1+equine CD14, equine TLR2+TLR1+CD14, or equine TLR2+TLR1+human CD14. The responses to Pam2CSK4 and Pam3CSK4 at TLR2/1 were unaffected by the presence of different species of CD14 (Figure 5B). Together, these data confirm that residues in both equine TLR2 and equine TLR1 are responsible for the low signal to Pam3CSK4 relative to Pam2CSK4.

## Discussion

In this study, we have demonstrated functionality of the equine TLR2/1 and TLR2/6 heterodimers, and shown the receptors respond to LTA in virtually the same way as those of human. We have also shown species differences are present in the responses of TLR2/1 to the synthetic bacterial lipopeptides Pam2CSK4 and Pam3CSK4, and that these differences are caused by both TLR2 and TLR1 but not CD14.

Comparison of the equine TLR2, -1 and -6 sequences with those of human and mouse show high sequence homology, which is highest in the TIR domain. TIR domains from different TLRs or TLR adaptor proteins are highly conserved between different species, which represents their evolutionary nature as detectors of conserved pathogenic products [26]. Molecular modelling suggests the TLR2, -1 and -6 ligand binding regions are predominantly conserved, with greatest amino acid variation at the periphery of the TLR2 ligand binding pocket and areas which would flank the ligand peptide groups. Evolutionary preservation of the TLR2 binding pocket is expected, since the majority of bacterial lipoproteins are triacylated or diacylated, and would presumably require insertion of two ester-bound acyl chains into the TLR2 pocket for receptor activation. Likewise, preservation of the TLR1 binding pocket is necessary to allow insertion of the third, amide-bound, acyl chain of triacylated lipopeptides. TLR6 is thought to have arisen by evolutionary adaptation of TLR1 for recognition of diacylated lipopeptides [27]. In the ligand-bound crystal structure of murine TLR2/6, two phenylalanines, F343 and F365, block the TLR6 binding pocket and prevent entry of a ligand acyl chain [1]. This confers diacylated ligand recognition only onto TLR6. These phenylalanines are conserved across all mammalian species (Additional file 6). Equine TLR6 is unable to recognise Pam3CSK4, which suggests the function of F343 and F365, to prevent entry of an acyl chain into the TLR6 binding pocket, is conserved in the horse. The reduced conservation of residues that constitute the ligand peptide-binding region in TLR2, -1 and -6 probably reflects the relative unimportance of this region in ligand binding specificity. The shape and size of the ligand peptide head group affects ligand potency at TLR2 in other species, due to effects on ligand positioning, however the interactions between receptors and ligand peptide groups play only a minor role in ligand binding [1]. The diversity of peptide groups between bacterial species may have created host selection pressure by altering requisite ligand sensitivity, leading to increased variation of this receptor region in response to species-specific pathogens.

All four pairs of equine/human TLR2/1 combinations signalled similarly to Pam2CSK4 (Figure 5A). Species specificity was seen with Pam3CSK4, with a partial, but not complete, restoration of the Pam3CSK4 response if either equine TLR2 or equine TLR1 was replaced with its human counterpart. It is unlikely the lowered Pam3CSK4 response is due to our L773W variant of equine TLR1, since replacement of equine TLR1 with human TLR1 should fully confer human TLR2/1 signalling to Pam3CSK4, which it does not. Amino acid differences between human and equine TLR2 and TLR1 may explain our species specificity data.

In the human TLR2/1/Pam3CSK4 crystal structure, G313 of human TLR1 forms a hydrogen bond with K3 of the ligand peptide head group, and replacement to S317 in equine TLR1 could interfere with this interaction due to a change in polarity [2]. There is also limited space for the ligand head group between TLR2 and TLR1, and so the increased size of the serine side chain could cause steric interference with ligand binding. A reduction in signalling, however, is not seen with Pam2CSK4, for which this interaction is also required. Absence of the ligand amide acyl chain could, however, be permissive of a subtle shift in ligand orientation that negates the effect of S317. This shift in orientation could also be favourable for Pam2CSK4 binding at equine TLR1, explaining the increased potency of the ligand at equine TLR2/1 relative to human.

Structural comparison of the human and murine TLR2 crystals bound to Pam3CSK4 and Pam2CSK4 respectively revealed small structural differences in the shape of the ligand binding pocket, which may be attributed to specific residues (human:murine L266F, P306L, T335L and L355F) [1]. The structural differences between the binding pockets are suggested to subtly affect acyl chain interaction, irrespective of presence or absence of a third ligand acyl chain. The former two residues are conserved between horse and mouse, the latter two between equine and human. The equine TLR2 binding pocket may therefore be expected to form an intermediate shape between that of human and mouse. Subtle differences between the equine TLR2/1 lipopeptide responses could then be attributed to the shape of the TLR2 binding pocket, as well as ligand interactions with TLR1. There is, however, significant permissible flexibility of the acyl chains within the human and murine TLR2 pockets, and so small alterations of the binding pocket shape should not grossly affect ligand responses [28]. The R357Q variant residue present in our equine TLR2 construct is unlikely to affect ligand behaviour selectively, since, for both diacylated and triacylated ligands, the TLR2 pocket contains the two ester-bound acyl chains of both ligands [1,2]. The considerable acyl chain flexibility within the pocket should therefore allow for some variability in chain conformation. Crystal structures of monomeric murine TLR2 bound to Pam3CSK4 and Pam2CSK4 also show identical conformations of the TLR2 pocket when the ligands are bound. LTA was equipotent at human and equine TLR2/1 and TLR2/6, therefore the shape and flexibility of the TLR2 pocket does not obviously affect binding to this ligand.

Our experiments with the equine TLRs were carried out in human cells and, theoretically, there could be differences in human MyD88 recruitment between human and equine TLRs. A failure of MyD88 recruitment is unlikely to explain our observed differential ligand effects at equine TLR2/1, since diacylated ligand recognition was highly similar for both species. It is, however, possible that a failure of Mal recruitment occurred, if equine recognition of triacylated ligands requires Mal and human does not. Murine TLR2 responses to Pam3CSK4 are variably Mal-independent in primary cells, yet Mal-dependent in immortalised cells, whereas responses to MALP-2 (a diacylated ligand) are Mal-dependent in both primary and immortalised cells [29-33]. The requirement by human TLR2 for Mal in triacylated ligand signalling is also unclear. Mal is required for signalling by human TLR2 to MALP-2, and is degraded following Pam3CSK4 stimulation of an immortalised human macrophage cell line in a manner similar to that following LPS stimulation [34,35]. The specific requirement for Mal in human TLR2 signalling to triacylated lipopeptides has not been shown definitively to the authors’ knowledge, however, and needs defining in human and equine primary cells to facilitate interpretation of our findings in the transfectant system.

Gram positive organisms frequently have diacylated lipopeptides due to lack of apo-lipoprotein N-acetyltransferase (Lnt) enzyme, which adds a third acyl chain [36,37]. This enzyme is usually present in Gram negative organisms, and so triacylated lipopeptides are more common in Gram negative species. Given the horse is very sensitive to LPS, it is tempting to speculate that a dampened TLR2 response to Gram negative organisms is evolutionarily advantageous, in order to prevent catastrophic immune responses to these pathogens [38]. The results of our study support this hypothesis, but further testing with other triacylated ligands, whole organisms and primary equine cells are required to substantiate this theory.

In conclusion, TLR2/1 and TLR2/6 heterodimers are likely to be important for bacterial detection in the horse, but the species differences we see in bacterial lipopeptide recognition may have important consequences for therapeutic drug design, such that human TLR2/1 antagonists may not be as useful in the horse and vice versa. Likewise, potential adjuvants designed to stimulate TLR2 may well not translate well from humans to horses.

## Competing interests

The authors declare that they have no competing interests.

## Authors’ information

KI and CEB are both MRCVS. CEB is a Reader in Immunopharmacology and Diplomate of the ECVPT.

## Supplementary Material

Additional file 1 **Expression of TLRs and responses to synthetic ligands of SW620 and HEK293 cells.** RT-PCR was performed on SW620 and HEK293 for TLR4 (lane 1), TLR2 (lane 2), TLR1 (lane 3), TLR6 (lane 4) and GAPDH (lane 5). **(A)** After 25 PCR cycles, TLR4 was detected in SW620, and TLR1 was detected in HEK293. **(B)** After 35 cycles, TLR2 was observed for both lines in the negative reaction (amplification of gDNA). TLR1 was observed in positive and negative reactions for SW620. A bright band was observed for TLR1 in HEK293 and not the negative control. A band was observed for TLR6 in the HEK293 not observed in the negative reaction, and observed at 30 cycles (not shown). A faint band appeared for TLR6 in SW620 not observed in the negative control or at 30 cycles (not shown). These findings indicate SW620 express TLR4, no TLR2, no TLR1 and possible low expression of TLR6. HEK293 express no TLR4, no TLR2, modest TLR1 and low TLR6. **(C)** SW620 were transiently transfected with human TLR2, TLR2+1 or TLR2+6, or no exogenous TLR, with human CD14 and reporters NF-κB-luc and *Renilla* luciferase, and stimulated 24 hours later with 20 ng/mL Pam2CSK4/Pam3CSK4 or medium alone. With exogenous TLR2, SW620 respond weakly to Pam2CSK4 and not to Pam3CSK4, consistent with low TLR6 expression. Exogenous TLR1 confers strong Pam3CSK4 signalling, and exogenous TLR1/6 confers strong Pam2CSK4 signalling. SW620 do not signal to these ligands without exogenous TLR. **(D)** HEK293 were transfected as SW620 and stimulated 48 hours later as SW620. HEK293 respond to both ligands with exogenous TLR2 only, consistent with endogenous TLR1/6 expression. Pam3CSK4 signalling is improved with exogenous TLR1. HEK293 do not signal to these ligands without exogenous TLR. Click here for file

Additional file 2 **Sequence identities of the equine receptor constructs with human and murine receptors.** Multiple sequence alignments for total receptor sequence, ectodomain only, and TIR domain only, reveal greatest conservation within the TIR domains. Click here for file

Additional file 3 **Sequence alignments of equine, human and murine TLR2 (A), TLR1 (B) and TLR6 (C).** Conserved residues across the three species are highlighted in green. Conservation is highest in the TIR domains for all three receptors, consistent with adaptor recruitment to this region. Conserved residues are spread evenly across the ectodomains and transmembrane domains. Click here for file

Additional file 4 **Variations between published equine receptor sequences and our constructs.** Models of the equine receptors were generated in SWISS-MODEL, based on the published human or murine crystal structures. **(A)** The equine TLR1 TIR structure (based on human TLR1 TIR; [PDB:1FYV]) shows W773 (green) lies on the opposing side of the TIR domain from the BB loop (yellow). **(B)** The equine TLR2 structure (based on human TLR2) shows Q357 lies somewhat away from the main dimerisation interface (yellow). Pam3CSK4 is shown in red. **(C)** The equine TLR6 structure (based on murine TLR6) shows I452 lies well away from the main dimerisation residues (yellow) and Pam2CSK4 binding site. Pam2CSK4 is shown in red. Click here for file

Additional file 5 **Crystal structure of human TLR2 and TLR1 [PDB code 2Z7X].** The TLR2 and TLR1 binding pockets are mostly lined with equine/human conserved residues (grey), whereas the regions surrounding the Pam3CSK4 (red) head group are mostly non-conserved (TLR2: blue; TLR1: green). Click here for file

Additional file 6 **Sequence alignment of TLR6.** The two phenylalanines that block the TLR6 binding pocket (highlighted green) are conserved across all domestic mammalian species. Click here for file
