# Supplementary figures and images for: The molecular basis for recognition of bacterial ligands at equine TLR2, TLR1 and TLR6
Source: Vet Res. 2013 Jul 4;44(1):50. doi: 10.1186/1297-9716-44-50 (PMC3716717; doi:10.1186/1297-9716-44-50)

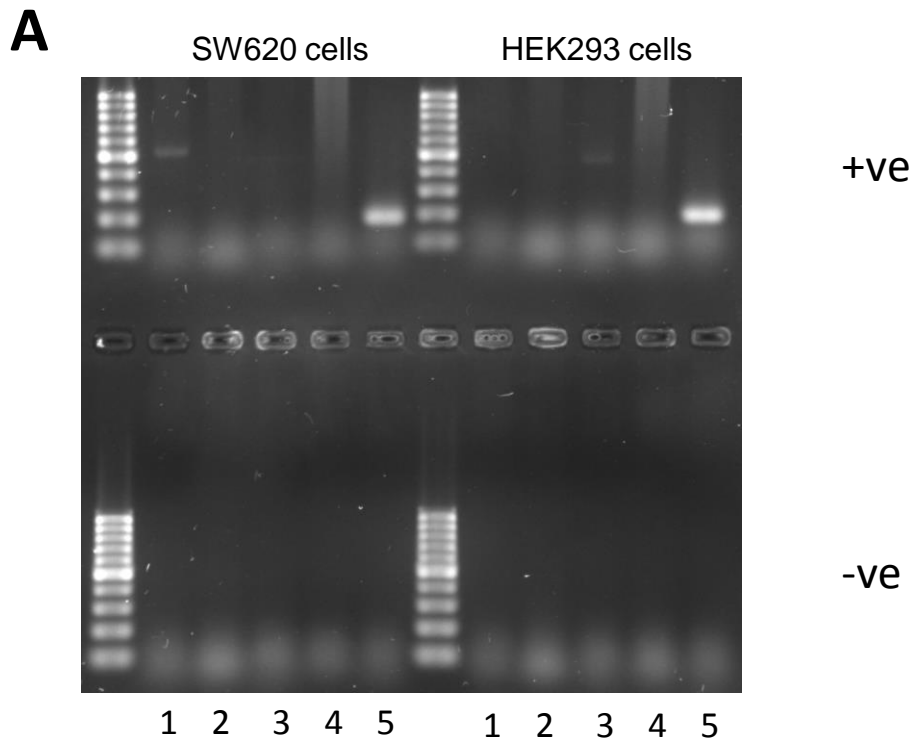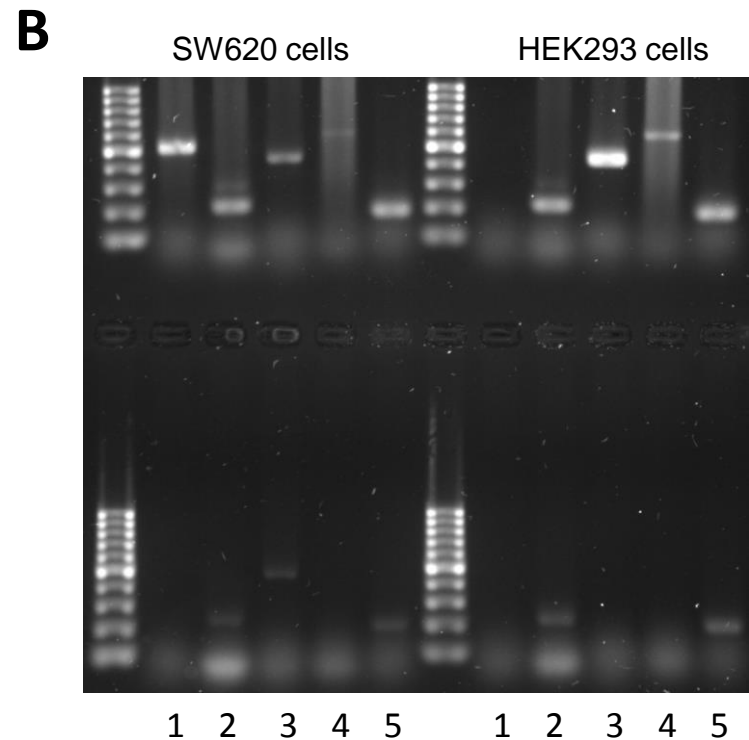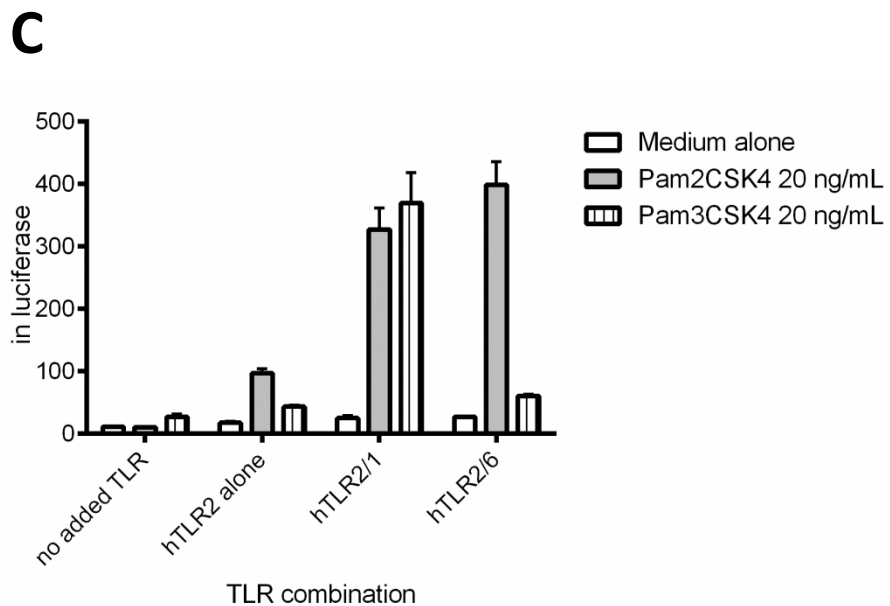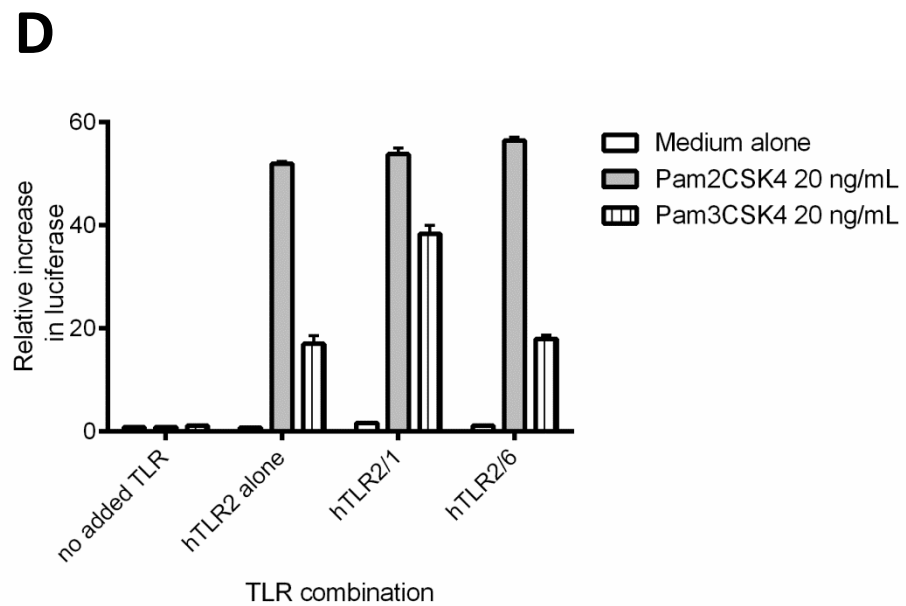

Supplement: Additional file 1 — Expression of TLRs and responses to synthetic ligands of SW620 and HEK293 cells. RT-PCR was performed on SW620 and HEK293 for TLR4 (lane 1), TLR2 (lane 2), TLR1 (lane 3), TLR6 (lane 4) and GAPDH (lane 5). (A) After 25 PCR cycles, TLR4 was detected in SW620, and TLR1 was detected in HEK293. (B) After 35 cycles, TLR2 was observed for both lines in the negative reaction (amplification of gDNA). TLR1 was observed in positive and negative reactions for SW620. A bright band was observed for TLR1 in HEK293 and not the negative control. A band was observed for TLR6 in the HEK293 not observed in the negative reaction, and observed at 30 cycles (not shown). A faint band appeared for TLR6 in SW620 not observed in the negative control or at 30 cycles (not shown). These findings indicate SW620 express TLR4, no TLR2, no TLR1 and possible low expression of TLR6. HEK293 express no TLR4, no TLR2, modest TLR1 and low TLR6. (C) SW620 were transiently transfected with human TLR2, TLR2+1 or TLR2+6, or no exogenous TLR, with human CD14 and reporters NF-κB-luc and Renilla luciferase, and stimulated 24 hours later with 20 ng/mL Pam2CSK4/Pam3CSK4 or medium alone. With exogenous TLR2, SW620 respond weakly to Pam2CSK4 and not to Pam3CSK4, consistent with low TLR6 expression. Exogenous TLR1 confers strong Pam3CSK4 signalling, and exogenous TLR1/6 confers strong Pam2CSK4 signalling. SW620 do not signal to these ligands without exogenous TLR. (D) HEK293 were transfected as SW620 and stimulated 48 hours later as SW620. HEK293 respond to both ligands with exogenous TLR2 only, consistent with endogenous TLR1/6 expression. Pam3CSK4 signalling is improved with exogenous TLR1. HEK293 do not signal to these ligands without exogenous TLR. [file 1297-9716-44-50-S1.pdf]

A

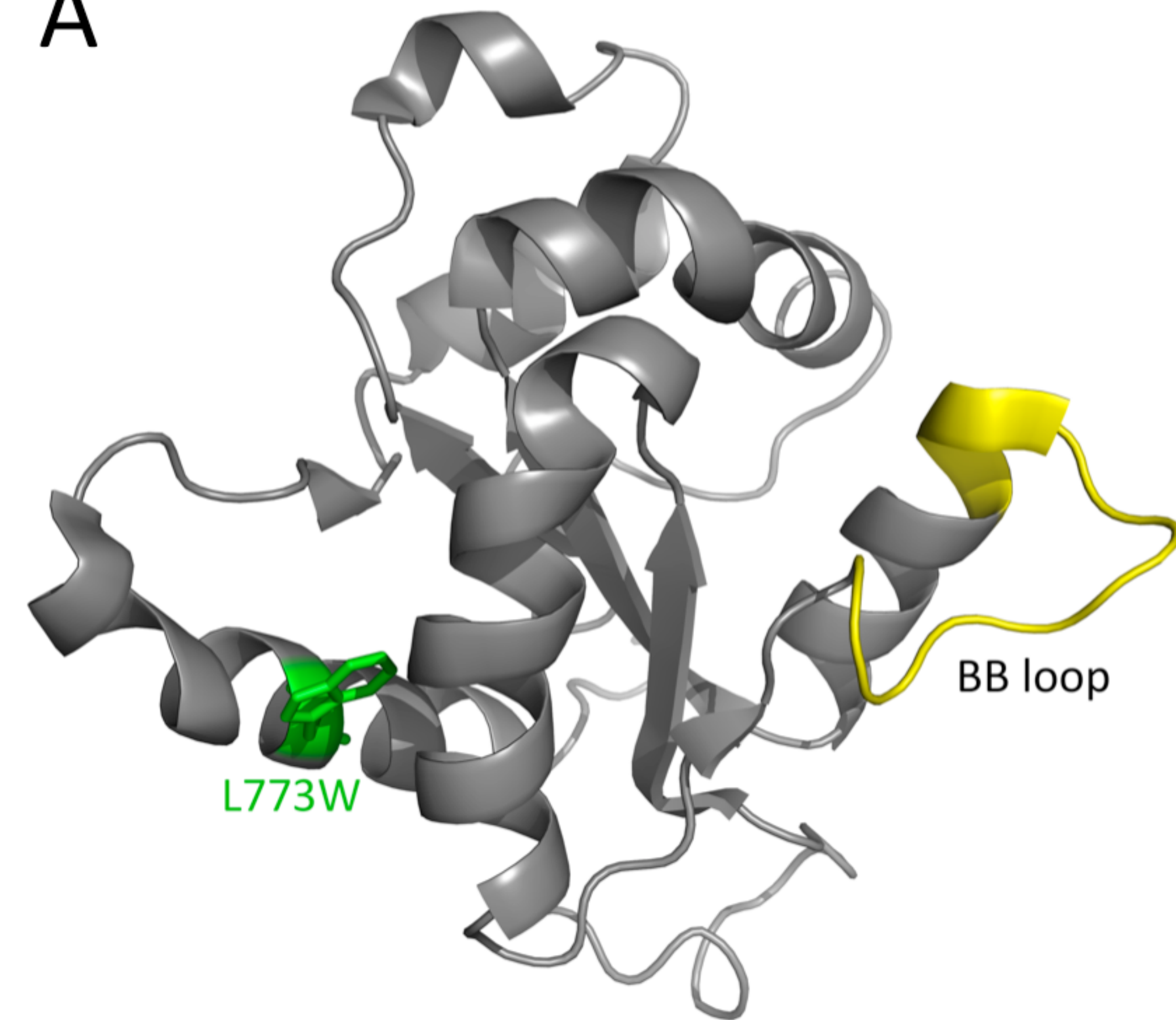

B

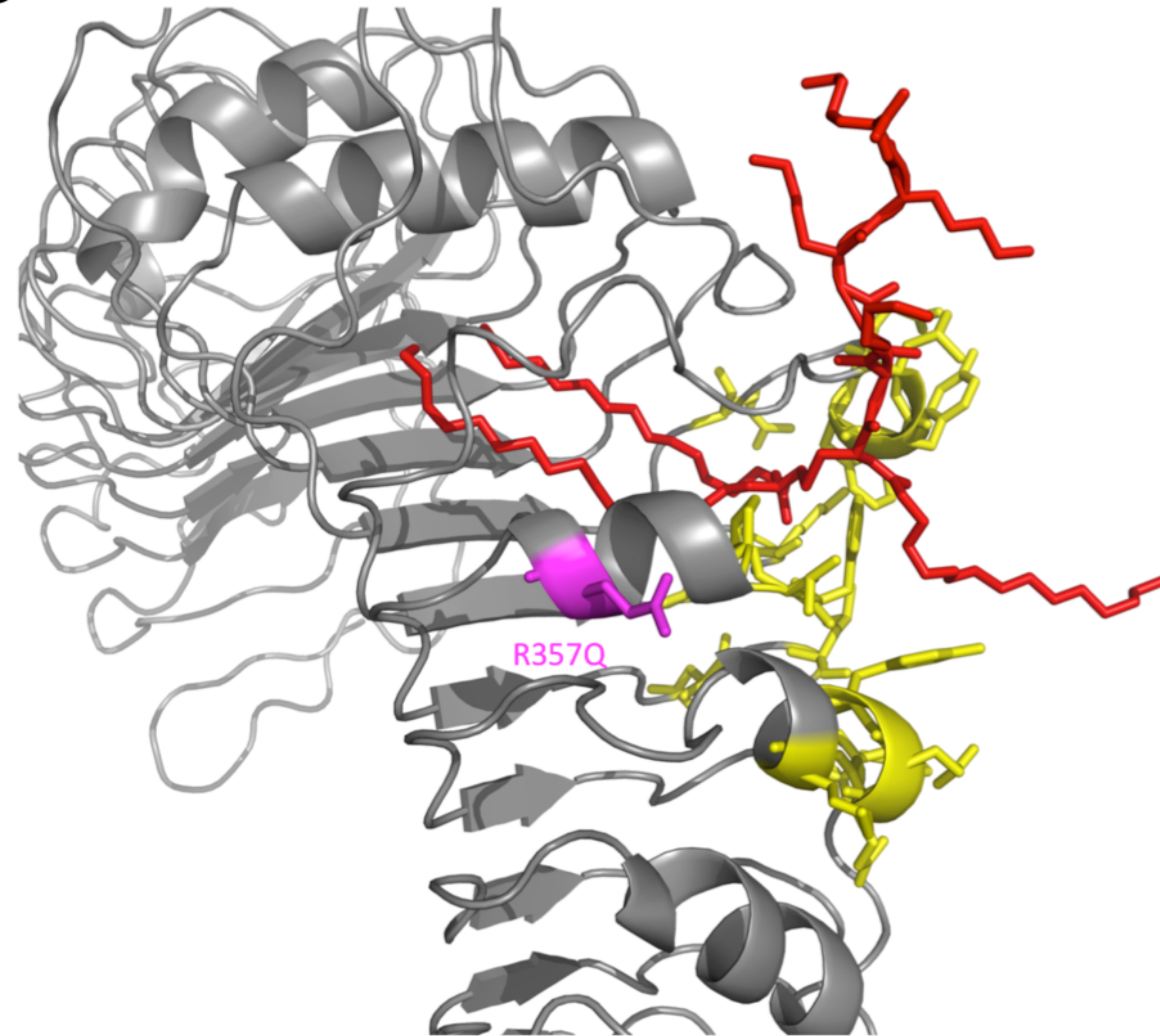

C

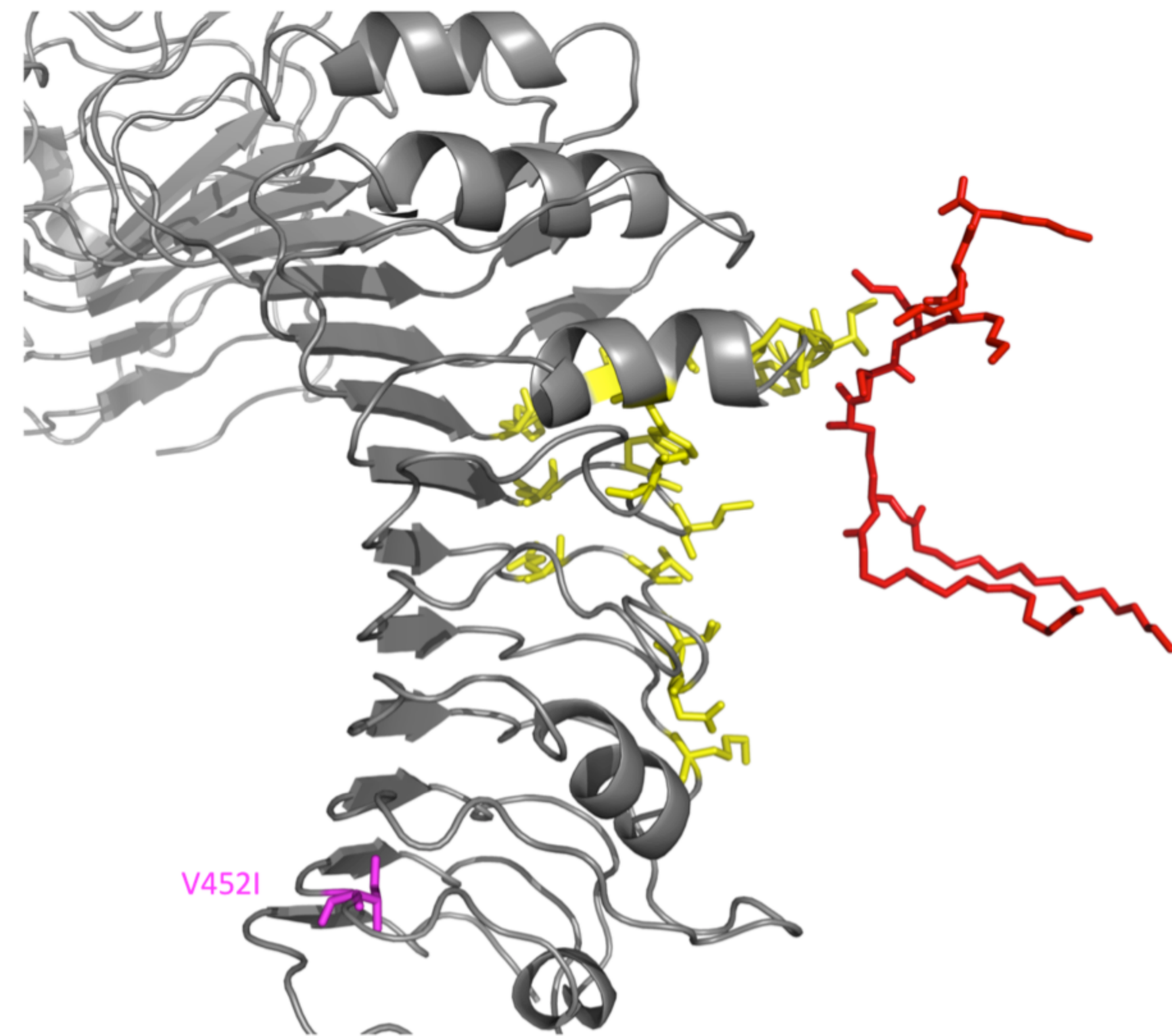

Supplement: Additional file 4 — Variations between published equine receptor sequences and our constructs. Models of the equine receptors were generated in SWISS-MODEL, based on the published human or murine crystal structures. (A) The equine TLR1 TIR structure (based on human TLR1 TIR; [PDB:1FYV]) shows W773 (green) lies on the opposing side of the TIR domain from the BB loop (yellow). (B) The equine TLR2 structure (based on human TLR2) shows Q357 lies somewhat away from the main dimerisation interface (yellow). Pam3CSK4 is shown in red. (C) The equine TLR6 structure (based on murine TLR6) shows I452 lies well away from the main dimerisation residues (yellow) and Pam2CSK4 binding site. Pam2CSK4 is shown in red. [file 1297-9716-44-50-S4.pdf]

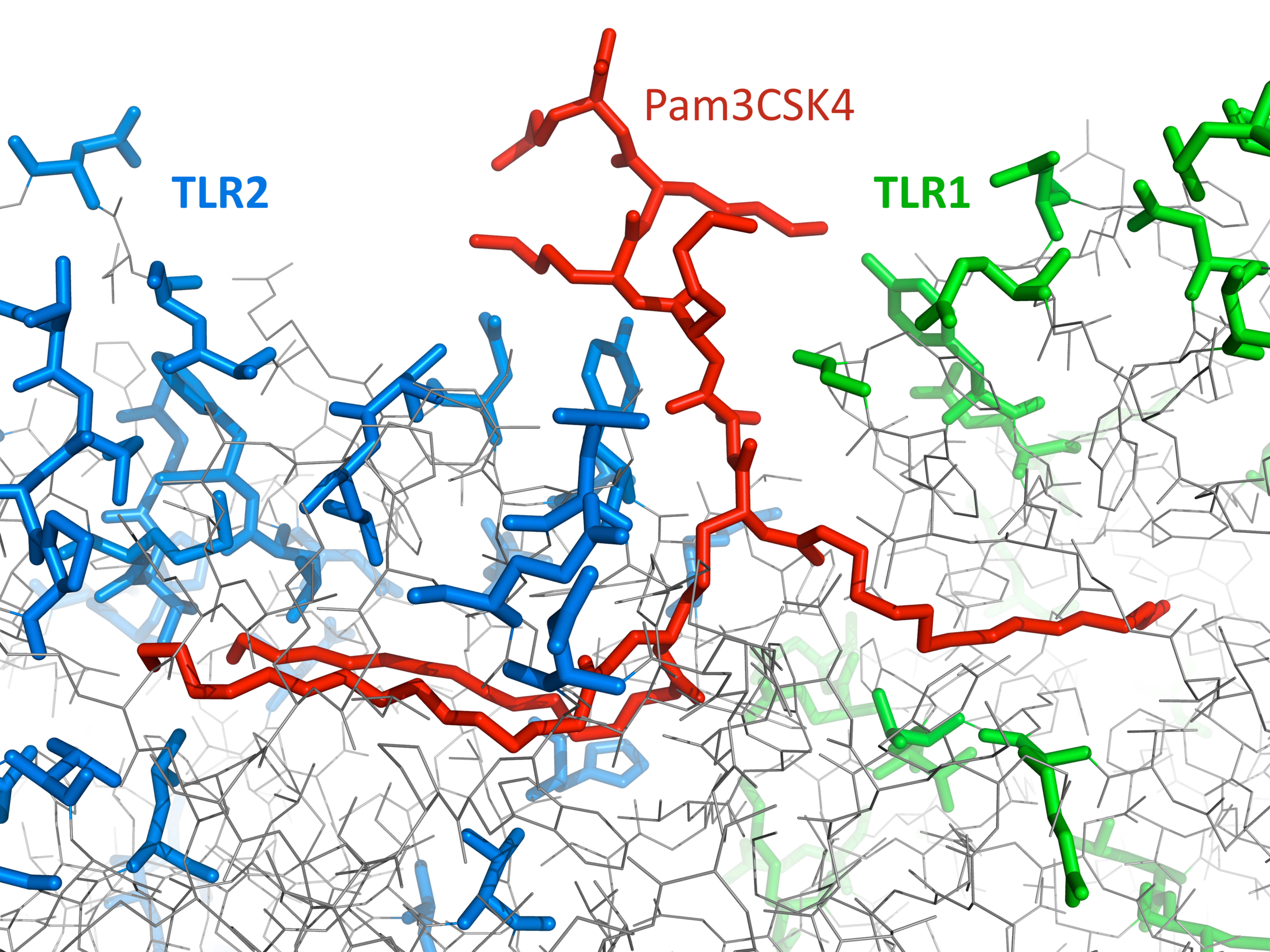

**TLR2**

**Pam3CSK4**

**TLR1**

Supplement: Additional file 5 — Crystal structure of human TLR2 and TLR1 [PDB code 2Z7X]. The TLR2 and TLR1 binding pockets are mostly lined with equine/human conserved residues (grey), whereas the regions surrounding the Pam3CSK4 (red) head group are mostly non-conserved (TLR2: blue; TLR1: green). [file 1297-9716-44-50-S5.pdf]
