# Supplementary material for: The molecular basis for recognition of bacterial ligands at equine TLR2, TLR1 and TLR6
Source: Vet Res. 2013 Jul 4;44(1):50. doi: 10.1186/1297-9716-44-50 (PMC3716717; doi:10.1186/1297-9716-44-50)
Supplement: Additional file 2 — Sequence identities of the equine receptor constructs with human and murine receptors. Multiple sequence alignments for total receptor sequence, ectodomain only, and TIR domain only, reveal greatest conservation within the TIR domains. [file 1297-9716-44-50-S2.pdf]

TLR2

Total identitiy

	Horse	Mouse
Human	81	70
Horse	-	69

Ectodomain identity

	Horse	Mouse
Human	78	65
Horse	-	63

TIR identity

	Horse	Mouse
Human	93	87
Horse	-	87

TLR1

Total identitiy

	Horse	Mouse
Human	81	73
Horse	-	71

Ectodomain identity

	Horse	Mouse
Human	79	70
Horse	-	68

TIR identity

	Horse	Mouse
Human	89	83
Horse	-	82

TLR6

Total identitiy

	Horse	Mouse
Human	80	73
Horse	-	71

Ectodomain identity

	Horse	Mouse
Human	77	71
Horse	-	68

TIR identity

	Horse	Mouse
Human	91	80
Horse	-	83
