# Supplementary material for: The molecular basis for recognition of bacterial ligands at equine TLR2, TLR1 and TLR6
Source: Vet Res. 2013 Jul 4;44(1):50. doi: 10.1186/1297-9716-44-50 (PMC3716717; doi:10.1186/1297-9716-44-50)
Supplement: Additional file 3 — Sequence alignments of equine, human and murine TLR2 (A), TLR1 (B) and TLR6 (C). Conserved residues across the three species are highlighted in green. Conservation is highest in the TIR domains for all three receptors, consistent with adaptor recruitment to this region. Conserved residues are spread evenly across the ectodomains and transmembrane domains. [file 1297-9716-44-50-S3.pdf]

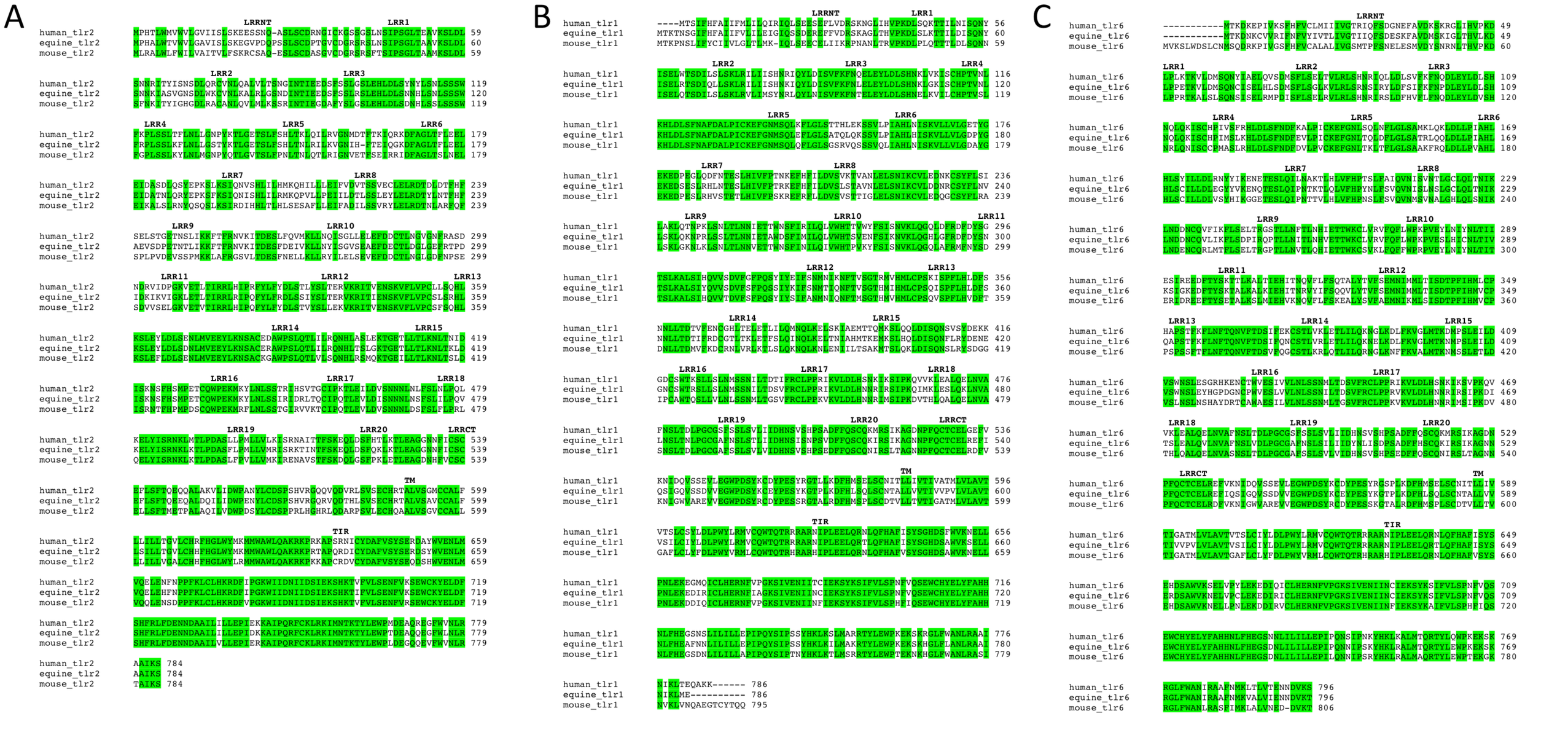
A
LRRNT
LRR1
human_tlr2 MPHTLWMVWVLGVIISLSKEESSNQ-ASLSCDRNGICKGSSGSLNSIPSGLTEAVKSLDL 59
equine_tlr2 MPHALWTVWVLGAVISLSKEGVPDQPSSLSCDPTGVCDGRSRSLNSIPSGLTAAVKSLDL 60
mouse_tlr2 MLRALWLFWILVAITVLFSKRCSAQ-ESLSCDASGVCDGRSRSFTSIPSGLTAAMKSLDL 59
LRR2
LRR3
human_tlr2 SNNRITYISNSDLQRCVNLQALVLTSNGINTIEEDSFSSLGSLEHLDLSYNYLSNLSSSW 119
equine_tlr2 SNNKIASVGNSDLWKCVNLKALRLGSNDINTIEEDSFSSLRSLEHLDLSNNHLSNLSSSW 120
mouse_tlr2 SFNKITYIGHGDLRACANLQVLMLKSSRINTIEGDAFYSLGSLEHLDLSDNHLSSLSSSW 119
LRR4
LRR5
LRR6
human_tlr2 FKPLSSLTFLNLLGNPYKTLGETSLFSHLTKLQILRVGNMDTFTKIQRKDFAGLTFLEEL 179
equine_tlr2 FRPLSSLKFLNLLGSTYKTLGETSLFSHLTNLRILKVGNIH-FTEIQGKDFAGLTFLEEL 179
mouse_tlr2 FGPLSSLKYLNLMGNPYQTLGVTSLFPNLTNLQTLRIGNVETFSEIRRIDFAGLTSLNEL 179
LRR7
LRR8
human_tlr2 EIDASDLQSYEPKSLKSIQNVSHLILHMKQHILLLEIFVDVTSSVECLELRDTDLDTFHF 239
equine_tlr2 EIDATNLQRVEPKSFKSIQNISHLILRMKQPVLLPEIILDTLSSLEYLELRDTYLNTFHF 239
mouse_tlr2 EIKALSLRNYQSQSLKSIRDIHHLTLHLSESAFLLEIFADILSSVRYLELRDTNLARFQF 239
LRR9
LRR10
human_tlr2 SELSTGETNSLIKKFTFRNVKITDESLFQVMKLLNQISGLLELEFDDCTLNGVGNFRASD 299
equine_tlr2 AEVSDPETNTLIKKFTFRNVKITDESFDEIVKLLNYISGVSEAEFDECTLDGLGEFRTPD 299
mouse_tlr2 SPLPVDEVSSPMKKLAFRGSVLTDESFNELLKLLRYILELSEVVFDDCTLNGLGDFNPSE 299
LRR11
LRR12
LRR13
human_tlr2 NDRVIDPGKVETLTIRRLHIPRFYLFYDLSTLYSLTERVKRITVENSKVFLVPCLLSQHL 359
equine_tlr2 IDKIKVIGKLETLTIRRLRIPQFYLFRDLSSIYSLTERVKRITIENSKVFLVPCSLSRHL 359
mouse_tlr2 SDVVSELGKVETVTIRRLHIFQFYLFYDLSTVYSLLEKVKRITVENSKVFLVPCSFSQHL 359
LRR14
LRR15
human_tlr2 KSLEYLDLSENLMVEEYLKNSACEDAWPSLQTLILRQNHLASLEKTGETLLTLKNLTNID 419
equine_tlr2 KSLEYLDLSDNLMVEEYLKNSACERAWPSLQTLILRQNHLTSLGKTGETLLTLKNLTKLD 419
mouse_tlr2 KSLEFLDLSENLMVEEYLKNSACKGAWPSLQTLVLSQNHLRSMQKTGEILLTLKNLTSLD 419
LRR16
LRR17
LRR18
human_tlr2 ISKNSFHSMPETCQWPEKMKYLNLSSTRIHSVTGCIPKTLEILDVSNNNLNLFSLNLPQL 479
equine_tlr2 ISKNSFHSMPETCQWPEKMKYLNLSSIRIDRLTQCIPQTLEVLDISNNNLNSFSLILPQV 479
mouse_tlr2 ISRNTFHPMPDSCQWPEKMRFLNLSSTGIRVVKTCIFQTLEVLDVSNNNLDSFSLFLPRL 479
LRR19
LRR20
LRRCT
human_tlr2 KELYISRNKLMTLPDASLLPMLLVLKISRNAITTFSKEQLDSFHTLKTLEAGGNNFICSC 539
equine_tlr2 KELYISRNKLKTLPDASFLPMLLVMRISRKTINTFSKEQLDSFQKLKTLEAGGNNFICSC 539
mouse_tlr2 QELYISRNKLKTLPDASLFPVLLVMKIRRENAVSTFSKDQLGSFPKLETLEAGDNHFVCSC 539
TM
human_tlr2 EFLSFTQEQQALAKVLIDWPANYLCDSPSHVRGQQVQDVRLSVSECHRTALVSGMCCALF 599
equine_tlr2 EFLSFTQEEQALDQILIDWPENYLCDSPSHVRGQRVQDTHLSVSECHRTALVSAVCCALF 599
mouse_tlr2 ELLSFTMETPALAQILVDWPDSYLCDSPPRLHGHRLQDARPSVLECHQAALVSGVCCALL 599
TIR
human_tlr2 LLILLTGVLCHRFHGLWYMKMMWAWLQAKRKFRKAPSRNICYDAFVSYSERDAYWVENLM 659
equine_tlr2 LSILLTGVLCHHFHGLWYMKMMWAWLQAKRKFRTAPQRDICYDAFVSYSERDSYWVENLM 659
mouse_tlr2 LLILLVGALCHHFHGLWYLRMMWAWLQAKRKFKKAPCRDVCYDAFVSYSEQDSHWVENLM 659
human_tlr2 VQELENFNPPFKLCLHKRDFIPGKWIIDNIIDSIEKSHKTVFVLSENFVKSEWCKYELDF 719
equine_tlr2 VQELEHFNPPFKLCLHKRDFIPGKWIIDNIIDSIEKSHKTIFVLSENFVRSEWCKYELDF 719
mouse_tlr2 VQQLENSDPPFKLCLHKRDFVPGKWIIDNIIDSIEKSHKTVFVLSENFVRSEWCKYELDF 719
human_tlr2 SHFRLFDENNDAAILILLEPIEKKAIPQRFCKLRKIMNTKTYLEWPMDEAQREGFWVNLR 779
equine_tlr2 SHFRLFDENNDAAILILLEPIDKKAIPQRFCKLRKIMNTKTYLEWPTDEAQQEGFWLNLR 779
mouse_tlr2 SHFRLFDENNDAAILVLLEPIERKAIPQRFCKLRKIMNTKTYLEWPLDEGQQEVFWVNLR 779
human_tlr2 AAIKS 784
equine_tlr2 AAIKS 784
mouse_tlr2 TAIKS 784
B
LRRNT
LRR1
human_tlr1 ----MTSIFHFAIIFMLILQIRIQLSEESEFLVDRSKNGLIHVPKDLSQKTTILNISQNY 56
equine_tlr1 MTKTNSGIFHFAIIFVLILEIGIQSSDEREFFVDRSKAGLTHVPKDLSLKTTILDISQNY 60
mouse_tlr1 MTKPNSLIFYCIIVLGLTLMK-IQLSEECELIIKRPNANLTRVPKDLPLQTTTLDLSQNN 59
LRR2
LRR3
LRR4
human_tlr1 ISELWTSDILSLSKLRILIISHNRIQYLDISVFKFNQELEYLDLSHNKLVKISCHPTVNL 116
equine_tlr1 ISELRTSDIQLLSKLRILIISHNKIQYLDISVFKFNLELEYLDLSHNKLGKISCHPTVNL 120
mouse_tlr1 ISELQTSDILSLSKLRVLIMSYNRLQYLNISVFKFNTELEYLDLSHNELKVILCHPTVSL 119
LRR5
LRR6
human_tlr1 KHLDLSFNAFDALPICKEFGNMSQLKFLGLSTTHLEKSSVLPIAHLNISKVLLVLGETYG 176
equine_tlr1 KHLDLSFNAFDALPICKEFGNMSQLEFLGLSATQLQKSSVLPIAHLHISKVLLVLGDPYG 180
mouse_tlr1 KHLDLSFNAFDALPICKEFGNMSQLQFLGLSGSRVQSSSVQLIAHLNISKVLLVLGDAYG 179
LRR7
LRR8
human_tlr1 EKEDPEGLQDFNTESLHIVFPTNKEFHFILDVSVKTVANLELSNIKCVLEDNKCSYFLSI 236
equine_tlr1 EKEDSESLRHLNTESLHIVFPTRKEFHFILDVSLSTAVNLELSNIKCVLDDNRCSYFLNV 240
mouse_tlr1 EKEDPESLRHVSTETLHIVFPSKREFRFLLDVSVSTTIGLELSNIKCVLEDQGCSYFLRA 239
LRR9
LRR10
LRR11
human_tlr1 LAKLQTNPKLSNLTLNNIETTWNSFIRILQLVWHTTWWYFSISNVKLQGQLDFRDFDYSG 296
equine_tlr1 LSKLQKNPRLSSLTLNNIETAWDSFIMILQLVWHTSVENFSIKNVKLQGHLGFRDFDYSN 300
mouse_tlr1 LSKLGKNLKLSNLTLNNVETTWNSFINILQIVWHTPVKYFSISNVKLQGQLAFRMFNYSD 299
LRR12
LRR13
human_tlr1 TSLKALSIHQVVSDVFGFPQSYIYEIFSNMNIKNFTVSGTRMVHMLCPSKISPFLHLDFS 356
equine_tlr1 TSLKALSIYQVVSDVFSFPQSSIYKIFSNMTIQNFTVSGTHMIHMLCPSQISPFLHLDFS 360
mouse_tlr1 TSLKALSIHQVVTDVFSFPQSYIYSIFANMNIQNFTMSGTHMVHMLCPSQVSPFLHVDFT 359
LRR14
LRR15
human_tlr1 NNLLTDTVFENCGHLTELETLILQMNQLKELSKIAEMTTQMKSLQQLDISQNSVSYDEKK 416
equine_tlr1 NNLLTDTIFRDCGTLTKLETFSLQINQLKELTNIAHMTKEMKSLHQLDISQNFLRYDENE 420
mouse_tlr1 DNLLTDMVFKDCRNLVRLKTLSLQKNQLKNLENITLTSAKMTSLQKLDISQNSLRYSDGG 419
LRR16
LRR17
LRR18
human_tlr1 GDCSWTKSLLSLNMSSNILTDTIFRCLPPRIKVLDLHSNKIKSIPKQVVKLEALQELNVA 476
equine_tlr1 GNCSWTRSLLSLNMSSNILTDSVFRCLPPRIKVLDLHNNRIRSIPKQIMKLESLQKLNVA 480
mouse_tlr1 IPCAWTQSLLVLNLSSNMLTGSVFRCLPFKVKVLDLHNNRIMSIPKDVTHLQALQELNVA 479
LRR19
LRR20
LRRCT
TM
human_tlr1 FNSLTDLPGCGSFSSLSVLIIDHNSVSHPSADFFQSCQKMRSIKAGDNPFQCTCELGEFV 536
equine_tlr1 LNSLTNLPGCGAFNSLSTLIIDHNSISNPSVDFFQSCQKIRSIKAGNNPFQCTCELREFI 540
mouse_tlr1 SNSLTDLPGCGAFSSLSVLVIDHNSVSHPSEDFFQSCQNIRSLTAGNNPFQCTCELRDFV 539
TIR
human_tlr1 KNIDQVSSEVLEGWPDSYKCDYPESYRGTLLKDFHMSELSCNITLLIVTIVATMLVLAVT 596
equine_tlr1 QSIGQVSSDVVEGWPDSYKCEYPESYKGTPLKDFHLSQLSCNTALLVVTIVVPVLVLAVT 600
mouse_tlr1 KNIGWVAREVVEGWPDSYRCDYPESSRGTALRDFHMSPLSCDTVLLTVTIGATMLVLAVI 599
human_tlr1 VTSLCSYLDLPWYLRMVCQWTQTRRARNIPLEELQRNLQFHAFISYSGHDSFWVKNELL 656
equine_tlr1 VSILCIYLDLPWYLRMVCQWTQTRRARNIPLEELQRTLQFHAFISYSGHDSAWVKSELL 660
mouse_tlr1 GAFLCLYFDLPWYVRMLCQWTQTRHRARHIPLEELQRNLQFHAFVSYSGHDSAWVKNELL 659
human_tlr1 PNLEKEGMQICLHERNFVPGKSIVENIITCIEKSYKSIFVLSPNFVQSEWCHYELYFAHH 716
equine_tlr1 PNLEKEDIRICLHERNFIAGKSIVENIINCIEKSYKSIFVLSPNFVQSEWCHYELYFAHH 720
mouse_tlr1 PNLEKDDIQICLHERNFVPGKSIVENIINFIEKSYKSIFVLSPHFIQSEWCHYELYFAHH 719
human_tlr1 NLFHEGSNSLILILLEPIPQYSIPSSYHKLKSLMARRTYLEWPKEKSKRGLFWANLRAAI 776
equine_tlr1 NLFHEAFNNLILILLEPIPQYSIPSSYHKLKILMAKRTYLEWPKEKSKHGLFLANLRAAI 780
mouse_tlr1 NLFHEGSDNLILILLAPIPQYSIPTNYHKLKTLMSRRTYLEWPTEKNKHGLFWANLRASI 779
human_tlr1 NIKLTEQAKK------ 786
equine_tlr1 NIKLME---------- 786
mouse_tlr1 NVKLVNQAEGTCYTQQ 795
C
LRRNT
human_tlr6 ----------MTKDKEPIVKSFHFVCLMIIIVGTRIQFSDGNEFAVDKSKRGLIHVPKD 49
equine_tlr6 ----------MTKDNKCVMRIFNFVYIVTLIVGTIIQFSDESKFAVDMSKIGLTHVLKD 49
mouse_tlr6 MVKSLWDSLCNMSQDRKPIVGSFHFVCALALIVGSMTPFSNELESMVDYSNRNLTHVPKD 60
LRR1
LRR2
LRR3
human_tlr6 LPLKTKVLDMSQNYIAELQVSDMSFLSELTVLRLSHNRIQLLDLSVFKFNQDLEYLDLSH 109
equine_tlr6 LPPETKVLDMSQNCISELHLSDMSFLSGLKVLRLSRNSIRYLDFSIFKFNPDLEYLDLSH 109
mouse_tlr6 LPPRTKALSLSQNSISELRMPDISFLSELRVLRLSHNRIRSLDFHVFLFNQDLEYLDVSH 120
LRR4
LRR5
LRR6
human_tlr6 NQLQKISCHPIVSFRHLDLSFNDFKALPICKEFGNLSQLNFLGLSAMKLQKLDLLPIAHL 169
equine_tlr6 NQLQKISCHPIMSLKHLDLSFNDFEVLPICKEFGNLTQLDFLGLSATRLQQLDLLPIAHL 169
mouse_tlr6 NRLQNISCCPMASLRHLDLSFNDFDVLPVCKEFGNLTKLTFLGLSAAKFRQLDLLPVAHL 180
LRR7
LRR8
human_tlr6 HLSYILLDLRNYYIKENETESLQILNAKTLHLVFHPTSLFAIQVNISVNTLGCLQLTNIK 229
equine_tlr6 HLSCILLDLEGYYVKENQTESLQIPNTKTLQLVFHPYNLFSVQVNISLNSLGCLQLTNIK 229
mouse_tlr6 HLSCILLDLVSYHIKGGETESLQIPNTTVLHLVFHPNSLFSVQVNMSVNALGHLQLSNIK 240
LRR9
LRR10
human_tlr6 LNDDNCQVFIKFLSELTRGSTLLNFTLNHIETTWKCLVRVFQFLWPKPVEYLNIYNLTII 289
equine_tlr6 LNDDNCQVLIKFLSDPIRQPTLLNITLNHVETTWKCLVRVFQFLWPKPVESLHICNLTIV 289
mouse_tlr6 LNDENCQRLMTFLSELTRGPTLLNVTLQHIETTWKCSVKLFQFFWPRPVEYLNIYNLTIT 300
LRR11
LRR12
human_tlr6 ESIREEDFTYSKTTLKALTIEHITNQVFLFSQTALYTVFSEMNIMMLTISDTPFIHMLCP 349
equine_tlr6 KSIGKEDFTYSKTALKALKIEHITNRVYIFSQQVLYTVFSEMNIMMLTISDTPFIHMVCP 349
mouse_tlr6 ERIDREEFTYSETALKSLMIEHVKNQVFLFSKEALYSVFAEMNIKMLSISDTPFIHMVCP 360
LRR13
LRR14
LRR15
human_tlr6 HAPSTFKFLNFTQNVFTDSIFEKCSTLVKLETLILQKNGLKDLFKVGLMTKDMPSLEILD 409
equine_tlr6 QAPSTFKFLNFTQNVFTDSIFQNCSTLVRLETLILQKNELKDLFKVGLMTKNMPSLEILD 409
mouse_tlr6 PSPSSFTFLNFTQNVFTDSVFQGCSTLKRLQTLILQRNGLKNFFKVALMTKNMSSLETLD 420
LRR16
LRR17
human_tlr6 VSWNSLESGRHKENCTWVESIVVLNLSSNMLTDSVFRCLPPRIKVLDLHSNKIKSVPKQV 469
equine_tlr6 VSWNSLEYHGPDGNCPWVESLVVLNLSSNILTDSVFRCLPPRVKVLDLHNNRIRSIPKDI 469
mouse_tlr6 VSLNSLNSHAYDRTCAWAESILVLNLSSNMLTGSVFRCLPPKVKVLDLHNNRIMSIPKDV 480
LRR18
LRR19
LRR20
human_tlr6 VKLEALQELNVAFNSLTDLPGCGSFSSLSVLIIDHNSVSHPSADFFQSCQKMRSIKAGDN 529
equine_tlr6 TSLEALQVLNVAFNSLVDLPGCGAFNSLSILIIDYNLISDPSADFFHSCQKIRSIKAGNN 529
mouse_tlr6 THLQALQELNVASNSLTDLPGCGAFSSLSVLVIDHNSVSHPSEDFFQSCQNIRSLTAGNN 540
LRRCT
TM
human_tlr6 PFQCTCELREFVKNIDQVSSEVLEGWPDSYKCDYPESYRCSPIKDFHMSELSCNITLLIV 589
equine_tlr6 PFQCTCELREFIQSIGQVSSDVVEGWPDSYKCEYPESYKGTPLKDFHLSQLSCNTALLVV 589
mouse_tlr6 PFQCTCELRDFVKNIGWVAREVVEGWPDSYRCDYPESSKGTAIRDFHMSPLSCDTVLLTV 600
TIR
human_tlr6 TIGATMLVLAVTVTSLCIYLDLPWYLRMVCQWTQTRRARNIPLEELQRNLQFHAFISYS 649
equine_tlr6 TIVVPVLVLAVTVSILCIYLDLPWYLRMVCQWTQTRRARNIPLEELQRTLQFHAFISYS 649
mouse_tlr6 TIGATMLVLAVTGAFLCLYFDLPWYVRMLCQWTQTRHRARHIPLEELQRNLQFHAFVSYS 660
human_tlr6 EHDSAWVKSELVPYLEKEDIQICLHERNFVPGKSIVENIINCIEKSYKSIFVLSPNFVQS 709
equine_tlr6 ERDSAWVKNELVPCLEKEDIRICLHERNFVPGKSIVENIINCIEKSYKSIFVLSPNFVQS 709
mouse_tlr6 EHDSAWVKNELLPNLEKDDIRVCLHERNFVPGKSIVENIINFIEKSYKAIFVLSPHFIQS 720
human_tlr6 EWCHYELYFAHHNLFHEGSNNLILILLEPIPQNSIPNKYHKLKALMTQRTYLQWPKEKSK 769
equine_tlr6 EWCHYELYFAHHNLFHEGSDNLILILLEPIPQNNIPSKYHKLRALMTQRTYLEWPKEKSK 769
mouse_tlr6 EWCHYELYFAHHNLFHEGSDNLILILLEPILQNNIPSRYHKLRALMAQRTYLEWPTEKGK 780
human_tlr6 RGLFWANIRAAFNMKLTLVTENNDVKS 796
equine_tlr6 RGLFWANIRAAFNMKVALVIENNDVKT 796
mouse_tlr6 RGLFWANLRASFIMKLALVNED-DVKT 806
