# Supplementary material for: The molecular basis for recognition of bacterial ligands at equine TLR2, TLR1 and TLR6
Source: Vet Res. 2013 Jul 4;44(1):50. doi: 10.1186/1297-9716-44-50 (PMC3716717; doi:10.1186/1297-9716-44-50)
Supplement: Additional file 6 — Sequence alignment of TLR6. The two phenylalanines that block the TLR6 binding pocket (highlighted green) are conserved across all domestic mammalian species. [file 1297-9716-44-50-S6.pdf]

TLR6 alignment

```
mouse          FLFSKEALYSVFAEMNIKMLSISDTPFIHMVCPPSPSSFTFLNFTQNVFTDSVFQGCSTL 387
rat            FLFVKDALYSVFAEMNIRMLTLSDTPFIHMVCPEFPSTFAFLNFTQNVFTDSIFQGCSTL 387
human          FLFSQTALYTVFSEMNIMMLTISDTPFIHMLCPHAPSTFKFLNFTQNVFTDSIFEKCSTL 376
chimp          FLFSQTALYTVFSEMNIMMLTISDTPFIHMLCPHAPSTFKFLNFTQNVFTDSIFEKCSTL 376
rhesus_monkey  FIFSQTALYTVFSEMNIMMLTISDTPFIHMLCPHAPSTFKFLNFTQNVFTDSIFEKCSTL 376
horse          YIFSQQVLYTVFSEMNIMMLTISDTPFIHMVCPQAPSTFKFLNFTQNVFTDSIFQNCSTL 376
dog            FLFSQTALYTIFSEMNIMMLTISDTPFIHMLCPPPSNTFKFLNFTQNVFTDSVFQSCSHL 419
cow            FIFSQTALYTVFSEMNILMLTISDTRFIHMLCPQEPSTFKFLNFTQNSFTDSVFQNCDTL 376
sheep          FIFSQTALYTVFSEMNIPMLTISDTPFIHMLCPQEPSTFKFLNFTQNAFTDSVFQNCDTL 377
pig            FIFSQTALYRVFSDMNIRMLTIADTHFIHMLCPQVPSTFNFLNFTQNVFTDSVFQNCKTL 376
```
